# Ginsenoside Rc ameliorated atherosclerosis *via* regulating gut microbiota and fecal metabolites

**DOI:** 10.3389/fphar.2022.990476

**Published:** 2022-09-15

**Authors:** Bin Xie, Xianpeng Zu, Zhicong Wang, Xike Xu, Guoping Liu, Runhui Liu

**Affiliations:** ^1^ School of Pharmacy, Naval Medical University, Shanghai, China; ^2^ Department of General Surgery, Xin Hua Hospital Affiliated to Shanghai Jiao Tong University School of Medicine, Shanghai, China

**Keywords:** atherosclerosis, gut microbiota, fecal metabolites, ginsenoside Rc, correlation

## Abstract

Atherosclerosis (AS) and the accompanied cardiovascular diseases (CVDs) were the leading cause of death worldwide. Recently, the association between CVDs, gut microbiota, and metabolites had aroused increasing attention. In the study, we headed our investigation into the underlying mechanism of ginsenoside Rc (GRc), an active ingredient of ginsenosides used for the treatment of CVDs, in apolipoprotein E-deficient (ApoE^−/−^) mice with high-fat diet (HFD). Seven-week-old male ApoE^−/−^ mice were randomly divided into four groups: the normal control (NC) group, the HFD group, the GRc group (40 mg/kg/d), and the atorvastatin (Ato) group (10 mg/kg/d). Atherosclerotic injury was evaluated by aortic lesions, serum lipid levels, and inflammatory factors. The composition of gut microbiota and fecal metabolite profile were analyzed using 16S rRNA sequence and untargeted metabolomics, respectively. The results showed that GRc significantly alleviated HFD-induced aortic lesions, reduced serum levels of total cholesterol (TC), triglyceride (TG), low-density lipoprotein cholesterol (LDL-C), tumor necrosis factor-α (TNF-α), and interleukin (IL)-6 and IL-1β, and increased high-density lipoprotein cholesterol (HFD-C) level, as well as the alteration of gut microbiota composition, function, and metabolite profile. GRc also reversed HFD change of Bacteroidetes and Firmicutes at the phylum level, Muribaculaceae, *Lactobacillus*, *Ileibacterium*, *Bifidobacterium*, *Faecalibaculum*, *Oscillibacter*, *Blautia*, and *Eubacterium_coprostanoligenes_group* at the genus level, and 23 key metabolites involved in taurine and hypotaurine metabolism, arginine biosynthesis, ATP-binding cassette (ABC) transporters, primary bile acid biosynthesis, purine metabolism, tricarboxylic acid (TCA) cycle, and glucagon signaling pathways. Additionally, eight differential intestinal floras at the genus level were associated with 23 key differential metabolites involving atherosclerotic injury. In conclusion, our results demonstrated that GRc ameliorated atherosclerotic injury, regulated microbial and metabolomic changes in HFD-induced ApoE^−/−^ mice, and suggested a potential correlation among gut microbiota, metabolites, and atherosclerotic injury regarding the mechanisms of GRc against AS.

## Introduction

Cardiovascular diseases (CVDs) are a group of disorders of the heart and blood vessels and the leading cause of death worldwide. Almost 17 million people died from CVDs in 2019, occupying 32% of global deaths (World Health Organization, 2021, https://www.who.int/news-room/fact-sheets/detail/cardiovascular-diseases-(cvds)). Atherosclerosis (AS), a systemic chronic inflammatory disease resulting in CVDs, is characterized by the formation of fibrofatty lesions in the artery wall (Genkel et al., 2020). The risk factors for AS and its thrombotic complications include low-density lipoprotein cholesterol (LDL-C), hypertension, cigarette smoking, obesity, and diabetes mellitus (Libby et al., 2019). Although the pathogenesis of AS is not totally clear, numerous investigations consider it a disorder of lipid metabolism, inflammation, and vascular endothelial damage (Libby et al., 2019). Despite the advanced treatment applied to clinic, such as lipid-lowering, anti-platelet, and anti-inflammatory drugs, there are therapeutic challenges including side effects and nonadherence to pharmacological therapy, which lead to most patients not meeting their desirable therapeutic goal (Libby et al., 2019). Therefore, the mortality resulting from AS and its complications is still high, and more advanced treatments for AS are urgent.

Accumulating studies indicate the role of gut microbiota and its metabolites on human diseases. Alterations in the composition of intestinal floras have been observed in a growing body of disorders including CVDs (Witkowski et al., 2020). Some microbial species from the intestine were found in the plaque of atherosclerotic patients, suggesting that gut microbiota might have a direct impact on AS (Fak et al., 2015). Recently studies showed that there was much more difference in the gut microbiota composition of patients with AS than healthy controls (Chen et al., 2021)^.^ In addition, the gut microbiota and its metabolites regulated inflammation, immunity, cholesterol, and lipid metabolism, which underlined the initiation and progression of AS (Jonsson and Backhed, 2017; Ma and Li, 2018). Various technologies for investigation of microbiota and its effects on host metabolism, such as bacterial 16S rRNA gene sequencing and metabolomics, allow further study of the relationship between gut metabolism and AS, as well as potential novel therapeutic targets.

**FIGURE 1:**
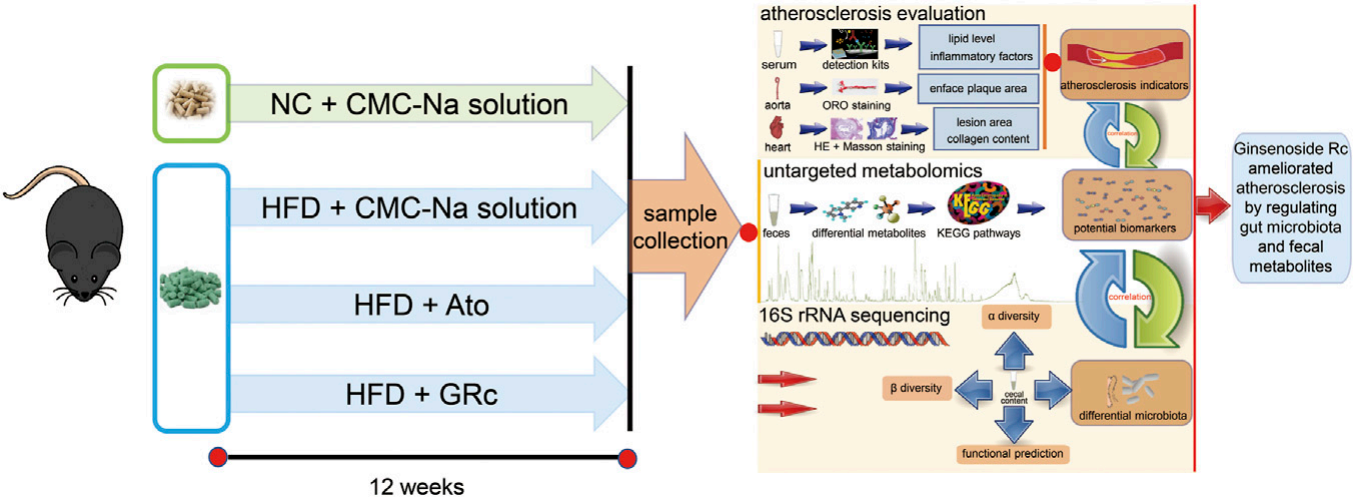
Workflow for examining the anti-atherosclerotic effects and potential mechanisms of GRc against AS in ApoE^−/−^ mice. Differential gut microbiota, fecal metabolites, and AS indexes were conjointly analyzed in the study.

*Panax ginseng* C.A. Meyer, a well-known medicinal herb, has been used for preventive and therapeutic purposes for thousands of years in Asian countries. Increasing studies reported the pharmacological activities and chemical components of *P. ginseng* (Mancuso and Santangelo, 2017; Wu et al., 2018). Ginsenosides, generally classified into protopanaxadiol and propanaxatriol due to the difference in C-6 chiral carbon substitution sites, are the main active components of *P. ginseng* used for the treatment of metabolic syndrome and CVDs (Fan et al., 2020; Im, 2020). Most Chinese patent medicines containing ginsenosides had been applied for AS, such as Shexiang Baoxin Pill, Yixingtongmai decoction, Tongxinluo, Xin-Ji-Er-Kang, and Tiaopi Huxin recipe (Zhang et al., 2022). Previous studies showed that protopanaxadiol-type ginsenoside Rd, Rb1, and Rg3 exhibited anti-atherosclerotic effects (Xue et al., 2021), and ginsenoside Rb1 could regulate gut microbiota and amino acid metabolism to improve HFD-induced insulin resistance in mice (Yang et al., 2021). A protopanaxadiol ginsenoside Rc (GRc) is an active ingredient of ginsenosides. Although it was poorly absorbed after oral administration in the host (Sun et al., 2014), it exhibited multiple pharmacological functions, including neuroprotective (Huang et al., 2021), anti-tumor (Zhu et al., 2021), anti-adipogenic (Yang and Kim, 2015), anti-inflammatory, and anti-oxidant (Shi et al., 2022) effects. However, the role of GRc against AS has not been investigated yet. Considering its poor bioavailability and chemical structure similar to Rd, Rb1, and Rg3, we hypothesized that GRc might have protective effects against AS and if so, the underlying mechanisms of anti-atherosclerotic effects could be probably associated with the regulation of gut microbiota and fecal metabolites. In this study, the role and potential mechanism of GRc against AS in HFD-induced ApoE^−/−^ mice were investigated for the first time using 16S rRNA sequence and untargeted fecal metabolomics methods (Figure 1). Based on the fact that GRc had a similar chemical structure to ginsenoside Rb1 which had anti-atherosclerotic effect and regulated gut microbiota composition, the study aimed to examine the anti-atherosclerotic effects and potential mechanisms of GRc against AS and provide a candidate small molecule for the prevention and treatment of CVDs.

## Materials and methods

### Reagents and materials

GRc (molecular weight = 1079.27, Lot: 20210419, CAS: 11021-14-0, and purity ≥98.0%) was purchased from Shanghai Sunny Biotech Co., Ltd. (Shanghai, China). Atorvastatin (Ato, Approval No. H20051408) was obtained from Pfizer Pharmaceuticals Ltd. (NY, United States). The kits for determining total cholesterol (TC), triglyceride (TG), LDL-C, and high-density lipoprotein cholesterol (HDL-C) were products of Nanjing Jiancheng Institute of Biological Engineering (Nanjing, China). The ELISA kits for mouse tumor necrosis factor-α (TNF-α), interleukin-6 (IL-6), and interleukin-1β (IL-1β) were provided by Neobioscience (Shenzhen, China).

### Animals and treatment

Seven-week-old male C57/BL6 ApoE^−/−^ mice of weight 23.7 ± 4 g (License Number: SCXK (Su) 2018-0008) were purchased from Jiangsu GemPharmatech Co., Ltd. (Jiangsu, China). All mice were maintained under a suitable environmental condition (22 ± 1°C, relative humidity of 60%, and 12 h/12 h light/dark cycle). All animal procedures were conducted according to the Guideline for the Care and Use of Laboratory Animals from Naval Medical University and approved by the Ethics Committee of Naval Medical University.

Mice were acclimated for 1 week and randomly assigned to four groups (*n* = 6): (1) the normal control (NC) group with a normal chow diet; (2) the high-fat diet (HFD) group containing 21% fat and 1.25% cholesterol (same volume of 0.5% sodium carboxymethyl cellulose solution by gavage); (3) the GRc group fed with HFD containing GRc (40 mg/kg /d GRc by gavage), and (4) the Ato group fed with HFD containing Ato (10 mg/kg/d Ato by gavage). GRc and Ato were dissolved in 0.5% sodium carboxymethyl cellulose solution, and all mice were fed once daily for 12 weeks consecutively. Body weight was monitored and recorded each week during the study.

At the end of the experiment, all animals were fasted overnight and anesthetized. Whole blood was drawn from the eyeball. Serum samples were isolated from whole blood through centrifugation at 2,500 *g* for 15 min at 4 °C and then immediately stored at −80 °C. Other organs or tissues were collected separately for further study.

### Assessment of atherosclerotic lesions

Atherosclerotic lesions were assessed with a protocol previously described (Lu et al., 2019), and the whole aorta from the arch to the iliac bifurcation was carefully removed and fixed in 4% paraformaldehyde solution overnight. Then, the aorta was washed three times with phosphate-buffered saline and stained with 0.5% Oil Red O (ORO) working solution. The extent of aortic AS was evaluated as the percentage of ORO-positive stained red area in relation to the area of the entire aorta luminal surface. Then, the hearts of mice were embedded in optimal cutting temperature compound and cut into 10-μm sections for histological analysis of atherosclerotic lesions in the aortic sinus. Measurement and quantification of the lesion area and the collagen content were carried out based on hematoxylin and eosin (H&E) and Masson’s trichrome staining, respectively. Representative images were analyzed using Image-Pro Plus 6.0 software (Media Cybernetics).

### Determination of serum biochemical parameters

Serum TC, TG, LDL-C, and HDL-C were measured using kits from Nanjing Jiancheng Bioengineering Institute following the manufacturer’s protocols. Serum systemic inflammatory cytokine levels of TNF-α, IL-6, and IL-1β were evaluated using ELISA kits according to the manufacturer’s instructions. The absorbance was measured at the corresponding wavelength using a Bio-Tek microplate reader (Winooski, VT, United States).

### 16S rRNA sequencing and analysis

Cecal contents were snap-frozen and stored at −80°C after collection. Genomic DNA was extracted from cecal contents using the MagPure Soil DNA LQ Kit (Magen) according to the manufacturer’s instructions. Concentration and quality of DNA were verified using a NanoDrop 2000 spectrophotometer (Thermo Fisher) and agarose gel electrophoresis, respectively. A thermocycler polymerase chain reaction (PCR) system (Bio-Rad) was applied for the amplification of the V3–V4 hypervariable parts of the bacterial 16S rRNA gene via primers 343F (5′-TACGGRAGGCAGCAG-3′) and 798R (5′- AGG​GTA​TCT​AAT-CCT-3′). The reverse primer contained a sample barcode, and both primers were connected with an Illumina sequencing adapter. The amplicon quality was visualized using gel electrophoresis. PCR products were purified with AMPure XP beads (Agencourt) and then quantified using a Qubit dsDNA assay kit (Life Technologies). Equal amounts of purified amplicon were pooled for subsequent sequencing.

Quality-filtration of raw FASTQ files was achieved via Trimmomatic (v0.35) followed by their merging through FLASH (v1.2.11). Reads with 75% of bases above Q20 were retained using QIIME software (v1.8.0). Then, reads with chimera were detected and removed using VSEARCH software (v2.4.2). Operational taxonomic units (OTUs) were grouped with a similarity cutoff of 97% utilizing VSEARCH, and the representative read of each OTU was selected using the QIIME (v1.8.0) package. All representative reads were annotated and blasted against the SILVA database (v132) using the RDP classifier with a confidence threshold of 70%. Microbial alpha (α) diversity in cecal contents was accessed by the indexes of Chao1 and Shannon. Beta (β) diversity was evaluated by principal coordinate analysis (PCoA) on an unweighted unifrac distance matrix. Linear discriminant analysis (LDA) coupled with effect size (LEfSe) measurements based on the non-parametric factorial Kruskal–Wallis sum-rank test and the Wilcoxon rank-sum test was used to identify taxa significantly different biomarkers between groups, with *p* < 0.05 and an LDA score threshold of 4. The phylogenetic investigation of communities by reconstruction of unobserved states (PICRUSt2) was applied to predict functional genes of the classified members of the microbiome through closed-reference-based OTU mapping against the Greengenes database (Langille et al., 2013). The 16S rRNA gene amplicon sequencing and analysis were conducted by OE Biotech Co., Ltd. (Shanghai, China).

### LC-MS-based untargeted metabolomics analysis

A volume of 60 mg fecal sample was transferred to a 1.5-ml Eppendorf tube. Two small steel balls were added to the tube. A volume of 24 μL internal standard (2-chloro-l-phenylalanine in methanol, 0.3 mg/ml) and extraction solvent with methanol/water (4/1, *v/v*) were added to each sample. QC samples were prepared by mixing aliquots of all samples to be a pooled sample. Samples were stored at −20°C for 5 min and then ground at 60 Hz for 2 min, ultrasonicated in cold water for 10 min, and stored at −20°C for 5 h. The extract was centrifuged at 13,000 g and 4°C for 10 min. A measure of 150 μl of supernatant from each tube was collected using crystal syringes, filtered through 0.22-μm microfilters, transferred to LC vials, and analyzed using a Dionex Ultimate 3000 RS UHPLC (Thermo Fisher Scientific) fitted with a Q-Exactive Plus Quadrupole-Orbitrap mass spectrometer equipped with a heated electrospray ionization (ESI) source. An ACQUITY UPLC HSS T3 (1.8 μm, 2.1 × 100 mm) was used, and the mobile phase consisted of acetonitrile and water containing 0.1% formic acid. Additionally, the following parameters were set: flow rate, 0.35 ml/min; column temperature, 45°C; and injection volume, 2 μl. The mass range was from *m/z* 100 to 1,000. The resolution was set at 70,000 for the full MS scans and 17,500 for HCD MS/MS scans. The collision energy was set at 10, 20, and 40 eV.

The original LC/MS data were processed using Progenesis QI (v2.3) software for baseline filtering, peak identification, integral, retention time correction, peak alignment, and normalization. Compound identification was based on the precise mass-to-charge ratio (*m/z*), secondary fragments, and isotopic distribution using the Human Metabolome Database (HMDB), LIPIDMAPS (v2.3), METLIN, EMDB, PMDB, and self-built databases to perform qualitative analysis. The matrix was imported in R to carry out principal component analysis (PCA) to observe the overall distribution among the samples and the stability of the whole analysis process. Partial least squares discriminant analysis (PLS-DA) was utilized to distinguish the metabolites that differ between groups. To prevent overfitting, 7-fold cross-validation and 200 response permutation testing were applied to evaluate the quality of the model. Variable importance in projection (VIP) scores was used to select differential metabolites between groups. Candidate metabolites having VIP > 1 and *p* < 0.05 were selected as potential biomarkers. Kyoto Encyclopedia of Genes and Genomes (KEGG) was applied for pathway annotation of differential metabolites.

### GC-MS-based untargeted metabolomics analysis

Metabolic profiling of the derivative sample was analyzed using an Agilent 7890B gas chromatography system (Agilent Technologies Inc., CA, United States) equipped with a DB-5MS fused silica capillary column (30 m × 0.25 mm × 0.25 μm). Helium (> 99.999%) was used as the carrier gas at a constant flow rate of 1 ml / min through the column. Then, parameter conditions were set as follows: injector temperature, 260°C; injection volume, 1 μl; MS quadrupole temperature, 150°C; ion source temperature, 230°C; collision energy, 70 eV; and mass range, 50–500 m/z. The QC samples were injected every 10 runs for data quality control.

The obtained GC/MS raw data were converted to an ABF format via Analysis Base File Converter software, followed by importing into MS-DIAL software for peak detection, deconvolution, alignment, and filtering. Metabolite characterization was based on the LUG database (untargeted database of GC-MS from Luming-bio). All internal standards and pseudo-positive peaks were removed. Then, the data matrix with three-dimensional datasets including sample information, peak names, and intensities was acquired for further analysis.

### Statistical analysis

Variability of serum biochemical parameters, plaque lesion area, the relative abundance of gut microbiota, and the segmented integration of metabolites were analyzed using GraphPad Prism 7 (GraphPad Software, Inc. United States). A part of the 16S rRNA analysis was carried out in R software. Metabolite cluster and Spearman correlation were analyzed using a cloud platform (https://cloud.oebiotech.cn/task/detail/correlation-multiomics-oehw/). The data were expressed as mean ± S.E.M. One-way ANOVA, *t*-test, Welch’s ANOVA, and Welch-corrected *t*-test were used according to data features. A *p*-value < 0.05 was considered statistically significant.

## Results

### Ginsenoside Rc reduced atherosclerotic lesions in high-fat diet-induced ApoE^−/−^ mice

**FIGURE 2:**
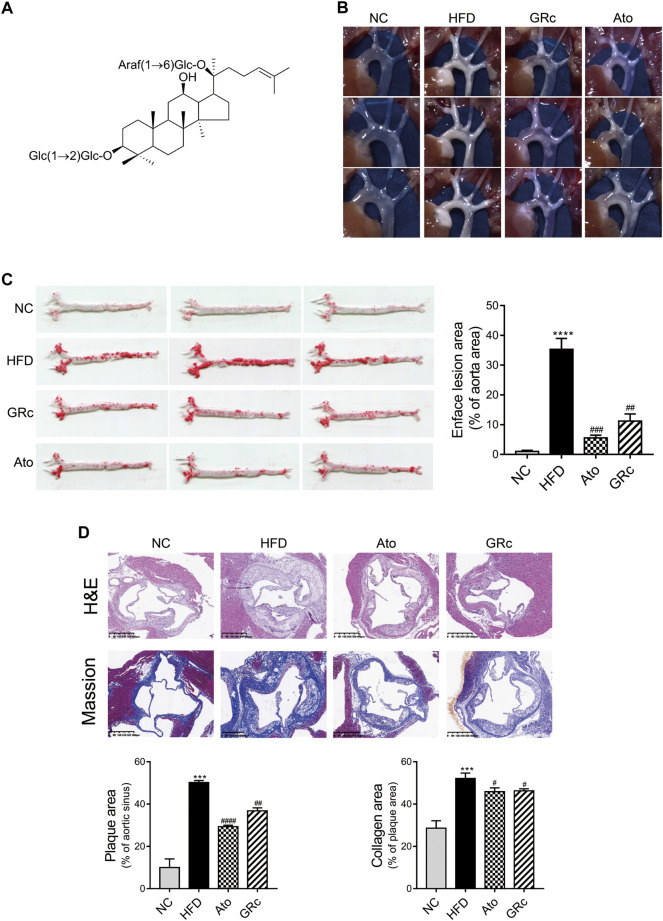
GRc alleviated atherosclerotic lesions in HFD-induced ApoE^−/−^ mice (*n* = 6). **(A)** Chemical structure of GRc. **(B)** Photographs of the aortic arch captured using the microscope. **(C)** Representative photographs of the whole aorta by Oil Red O staining, and the plaque area of the whole aorta was quantitated. **(D)** Representative images of aortic sinus sections by H&E staining and Masson’s staining; bar = 400 μm. The lesion and collagen area in the aortic sinus were quantitated. The data are expressed as means ± SEM. *p < 0.05, **p < 0.01, and ***p < 0.001 vs. NC; #*p* < 0.05, ##*p* < 0.01, and ###*p* < 0.001 vs. HFD. NC, normal control; HFD, high-fat diet; Ato, atorvastatin; GRc, ginsenoside Rc.

The chemical structure of GRc is shown in Figure 2A. During the period of the animal experiment, the body weight of mice was monitored to investigate the effects of GRc on the physiological characteristics. As shown in Supplementary Figure S1, the weight gain showed no significant difference in NC, HFD, Ato, and GRc groups during treatment periods, implying that the dose of GRc in the study was safe for HFD-induced ApoE^−/−^ mice.

HFD-induced ApoE^−/−^ mice have been widely used to construct the animal model of AS (Zhang et al., 2021). The effect of GRc on AS was determined by the size of plaque lesions in the entire aorta of HFD-induced ApoE^−/−^ mice. The atherosclerotic plaque area in the aorta of the HFD group had a significant increase compared with that of the NC group. Compared with the HFD group, GRc or Ato significantly reduced the aortic plaque area in HFD-induced ApoE^−/−^ mice (Figures 2B,C). Then, the atherosclerotic lesions and collagen content in the aortic sinus were measured and quantified using H&E and Masson’s trichrome staining. The size of atherosclerotic lesions and collagen content in the aortic sinus were much larger in the HFD group than the NC group. Compared with the HFD group, GRc or Ato treatment apparently reduced the size of the atherosclerotic lesion area and collagen content in the aortic sinus (Figure 2D), demonstrating that GRc treatment was similar to Ato regarding the reduction of HFD-induced atherosclerotic injury.

### Ginsenoside Rc improved lipid levels and systemic inflammation in ApoE^−/−^ mice fed with high-fat diet

**FIGURE 3:**
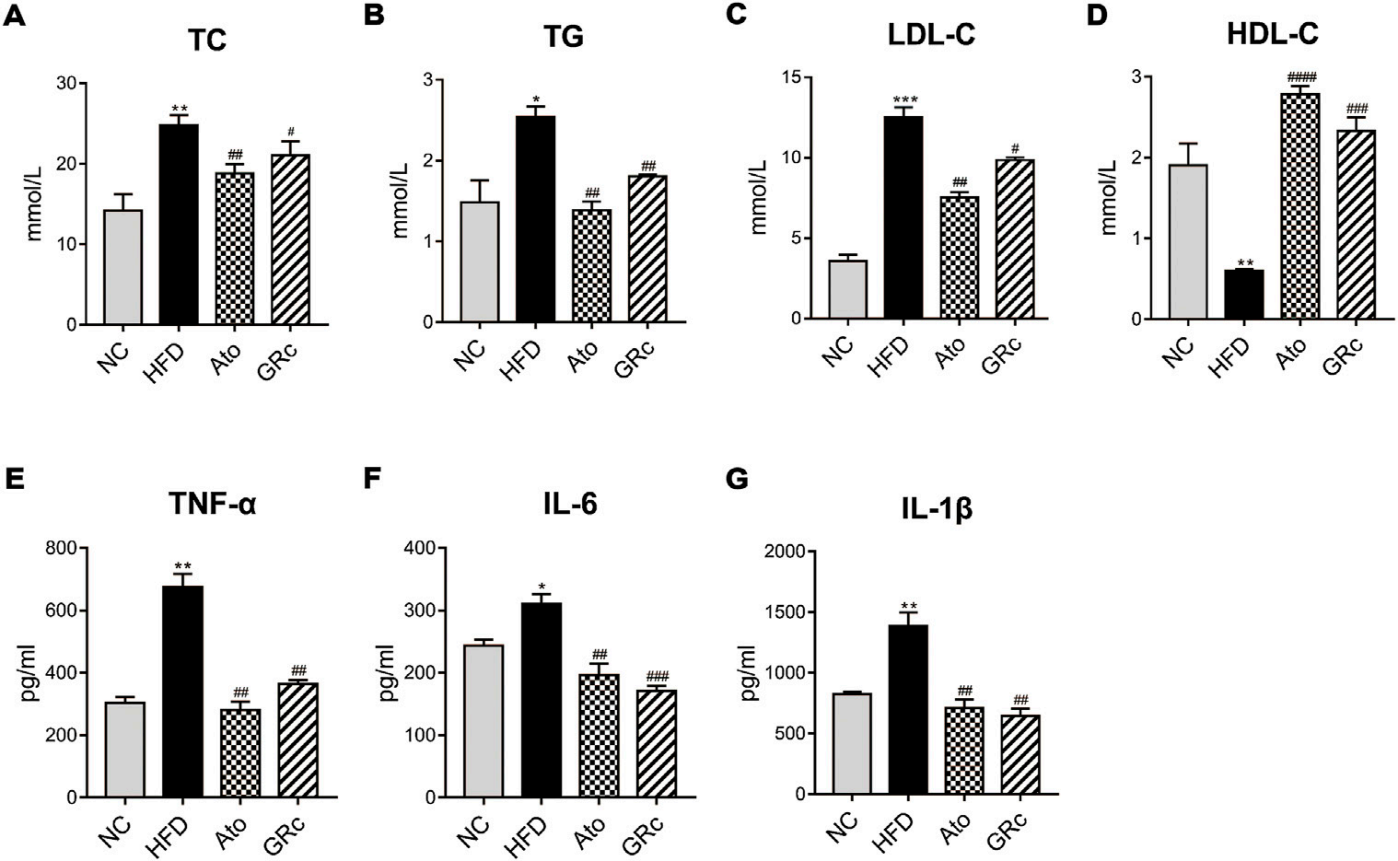
GRc regulated lipid and systemic inflammatory cytokines in HFD-induced ApoE^−/−^ mice. **(A–D)** Levels of serum lipids (TC, TG, LDL-C, and HDL-C) per group. **(E–G)** Levels of inflammatory cytokines (TNF-α, IL-6, and IL-1β) per group. The data are expressed as means ± SEM. *p < 0.05, **p < 0.01, and ***p < 0.001 vs. NC; #*p* < 0.05, ##*p* < 0.01, and ###*p* < 0.001 vs. HFD. NC, normal control; HFD, high-fat diet; Ato, atorvastatin; GRc, ginsenoside Rc.

Given that lipid levels and systemic inflammatory cytokines play an important role in the progress of AS, we further determined the levels of serum TC, TG, LDL-C, HDL-C, TNF-α, IL-6, and IL-1β. Compared with the NC group, serum TC, TG, and LDL-C in the HFD group significantly increased, while serum HDL-C was significantly decreased. Treatment with GRc or Ato significantly decreased TC, TG, and LDL-C levels and increased HDL-C levels (Figures 3A–D). As shown in Figures 3E–G, serum TNF-α, IL-6, and IL-1β were much higher in ApoE^−/−^ mice fed with HFD than the NC group. Both GRc and Ato groups markedly reduced inflammatory cytokines TNF-α, IL-6, and IL-1β. These results indicated that GRc improved lipid levels and systemic inflammation in HFD-fed ApoE^−/−^ mice.

### Effects of ginsenoside Rc on the composition of gut microbiota in ApoE^−/−^ mice

**FIGURE 4:**
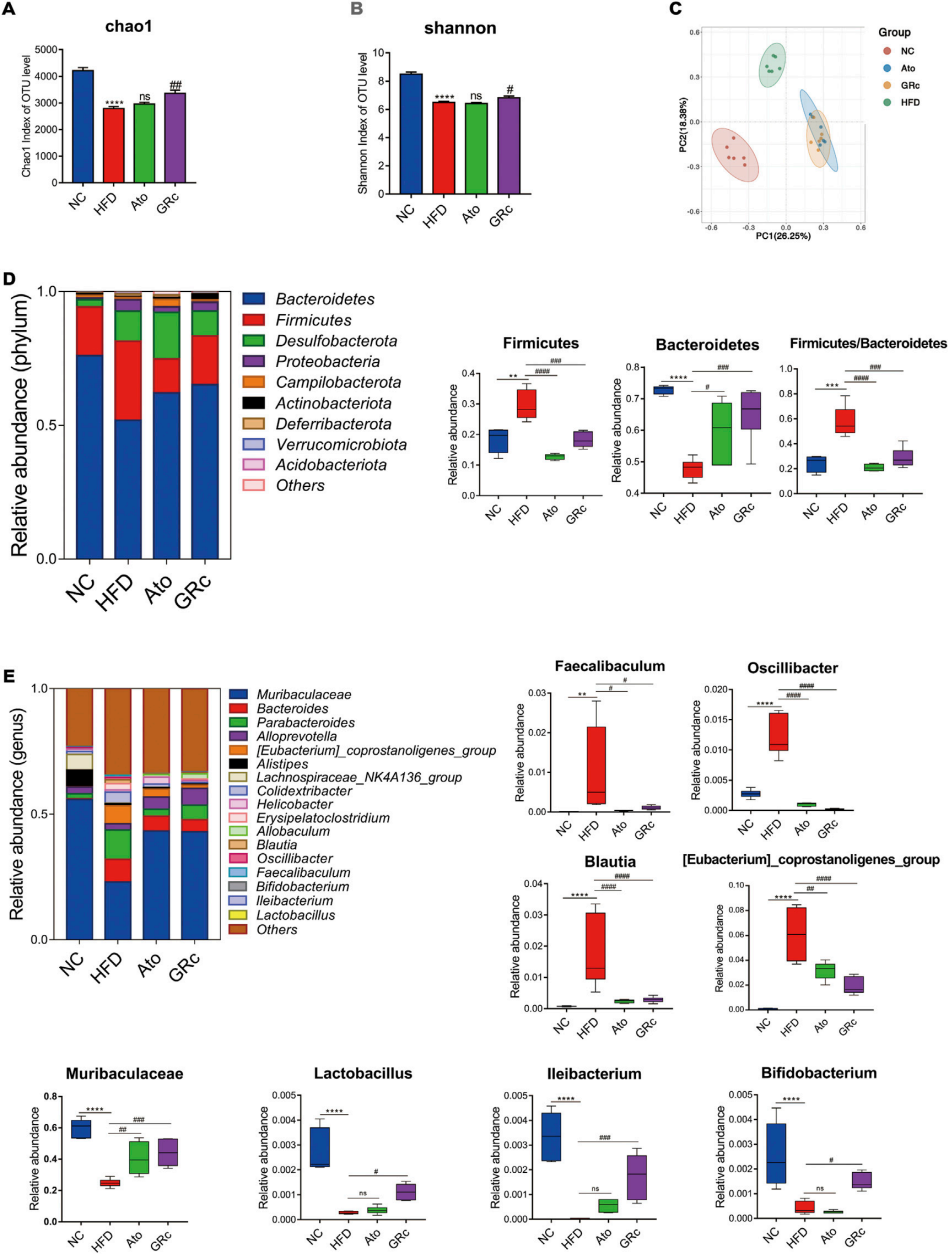
Effects of GRc on the composition of gut microbiota in HFD-induced ApoE^−/−^ mice (*n* = 6). **(A–B)** Richness and diversity of the gut microbiome were assessed by Chao1 and Shannon indexes, respectively. **(C)** PCoA analysis based on unweighted unifrac metrics for all samples at the OUT level. **(D)** Structure and relative abundance of Firmicutes and Bacteroidetes at the phylum level and the ratio of Firmicutes/Bacteroidetes. **(E)** Structure and relative abundance of Muribaculaceae, *Eubacterium_coprostanoligenes_group*, *Faecalibaculum*, *Oscillibacter*, *Blautia*, *Ileibacterium*, *Lactobacillus*, and *Bifidobacterium* at the genus level. The data are expressed as means ± SEM. *p < 0.05, **p < 0.01, and ***p < 0.001 vs. NC; #*p* < 0.05, ##*p* < 0.01, and ###*p* < 0.001 vs. HFD; ns, not significant; NC, normal control; HFD, high-fat diet; Ato, atorvastatin; GRc, ginsenoside Rc.

Sequencing of 16S rRNA was conducted to determine the effect of GRc treatment on gut microbiota composition. Overall, a total of 1,741,378 clean reads were obtained from 24 cecal samples (72,557.42 ± 281.66 clean reads per sample), and 1,506,396 valid tags with average lengths ranging from 415.83 to 419.56 were observed. After data processing, a total of 9,802 OTUs (2,414.13 ±75.93 OTUs per sample) were obtained. To further observe community richness and diversity, intestinal microbial richness was accessed by the Chao1 index and intestinal microbial diversity by the Shannon index. The curves within each sample were flat in the Shannon rarefaction map (Supplementary Figure S2), implying that the range of sequencing was able to determine the biodiversity of bacterial communities. As shown in Figures 4A,B, Chao1 and Shannon indexes in the HFD group significantly decreased compared with those in the NC group, indicating that there were markedly decreased intestinal microbial richness and diversity after HFD intake in ApoE^−/−^ mice. PCoA based on unweighted unifrac metrics was used to demonstrate the difference in the composition of the bacterial communities. PCoA spots in the same group were well clustered, implying that there was a similar composition of the bacterial communities among samples from the same group. The distance of PCoA spots in NC, HFD, and GRc groups was apparently separated, which suggested different constructures of the bacterial communities among the groups. The GRc-related difference was represented by PC1, which explained 26.25% of the total variation in the microbial composition. The HFD-related difference was represented by PC2, which explained 18.38% of the total variation (Figure 4C). The PCoA results showed that GRc treatment contributed more to gut microbiota structure than HFD feeding alone.

The gut microbiota significantly changed at different taxonomic levels among the groups as shown in Supplementary Figure S3. The differential intestinal floras related to CVDs were listed. At the phylum level, the top six microflora of mice were Bacteroidetes, Firmicutes, Desulfobacterota, Proteobacteria, Campilobacterota, and Actinobacteriota, the composition of which differed apparently among NC, HFD, and GRc groups. The abundance of Bacteroidetes in the HFD group was much lower than that in the NC group, while GRc or Ato treatment reversed the change. Compared with the NC group, the HFD group had a markedly increased abundance of Firmicutes, which was significantly decreased in the GRc or Ato group. The aforementioned results lead to a higher ratio of Firmicutes to Bacteroidetes in the HFD group than that in the NC group, and a lower ratio in the GRc or Ato group than that in the HFD group (Figure 4D). At the genus level, the relative abundance of *Faecalibaculum*, *Oscillibacter*, *Eubacterium_coprostanoligenes_group*, and *Blautia* was much higher, while the relative abundance of Muribaculaceae, *Lactobacillus*, *Ileibacterium*, and *Bifidobacterium* markedly reduced after HFD feeding. The imbalance of intestinal floras was recovered to some extent by GRc treatment which reversed the effects of HFD at the genus level of microbiota composition. The Ato group exhibited no significant effects on *Ileibacterium*, *Bifidobacterium*, and *Lactobacillus* (Figure 4E). Overall, GRc treatment could regulate specific intestinal flora at different taxonomic levels.

**FIGURE 5:**
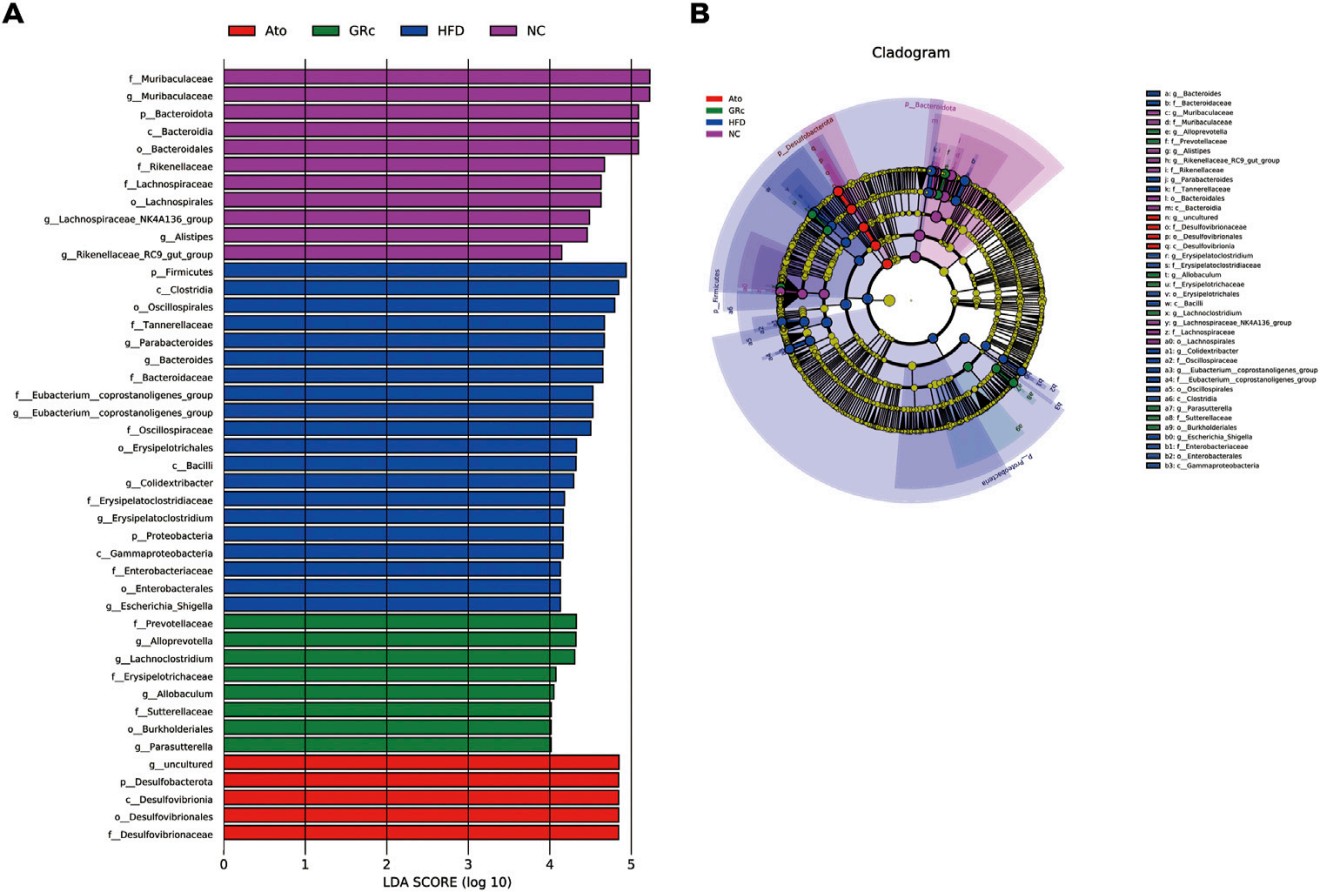
Core bacterial phenotype analysis in HFD-induced ApoE^−/−^ mice (*n* = 6). **(A)** LDA scores of taxa enriched at different taxonomy levels (LDA significant threshold = 4). **(B)** Taxonomic cladogram generated by LEfSe analysis showing taxa significantly enriched in the NC group (purple), HFD group (blue), GRc group (green), and Ato group (red), respectively. Each ring represents a taxonomic level from phylum to genus. The diameter of each dot on the ring represents the relative abundance of the taxon. NC, normal control; HFD, high-fat diet; Ato, atorvastatin; GRc, ginsenoside Rc.

In addition, the LEfSe analysis was used for highlighting the core bacterial phenotypes from the phylum to genus contributing to the variations in the microbiota composition. As shown in Figures 5A,B, the NC group was enriched with the phylum Bacteroidetes, the class Bacteroidia, the order Bacteroidales and Lachnospiraceae, and the family Muribaculaceae (from family to genus), Rikenellaceae, and Lachnospiraceae, as well as the genus *Alistipes*, Rikenellaceae*_RC9_gut_group*, and Lachnospiraceae*_NK4A136_group* , while the HFD group was enriched with the phylum Firmicutes and Proteobacteria, the class Clostridia, Bacilli, and Gammaproteobacteria, the order Oscillospirales, Erysipelotrichales and Enterobacterales, the family Tannerellaceae, Bacteroidaceae, Eubacterium_coprostanoligenes_group (from family to genus), Oscillospiraceae, Erysipelatoclostridiaceae, and Enterobacteriaceae, and the genus *Parabacteroides*, *Bacteroides*, *Colidextribacter*, *Erysipelatoclostridium*, and *Escherichia_Shigella*. The aforementioned results indicated that HFD feeding reduced the gut microbiota enriched in the NC group. The gut microbiota enriched in the GRc group were the order Burkholderiales, the family Prevotellaceae, Erysipelotrichaceae, and Sutterellaceae, and the genus *Alloprevotella*, *Lachnoclostridium*, *Allobaculum*, and *Parasutterella*, while intestinal flora in the Ato group was enriched at the lowest level.

### Predictive functional profiling of microbial communities by PICRUSt2

**FIGURE 6:**
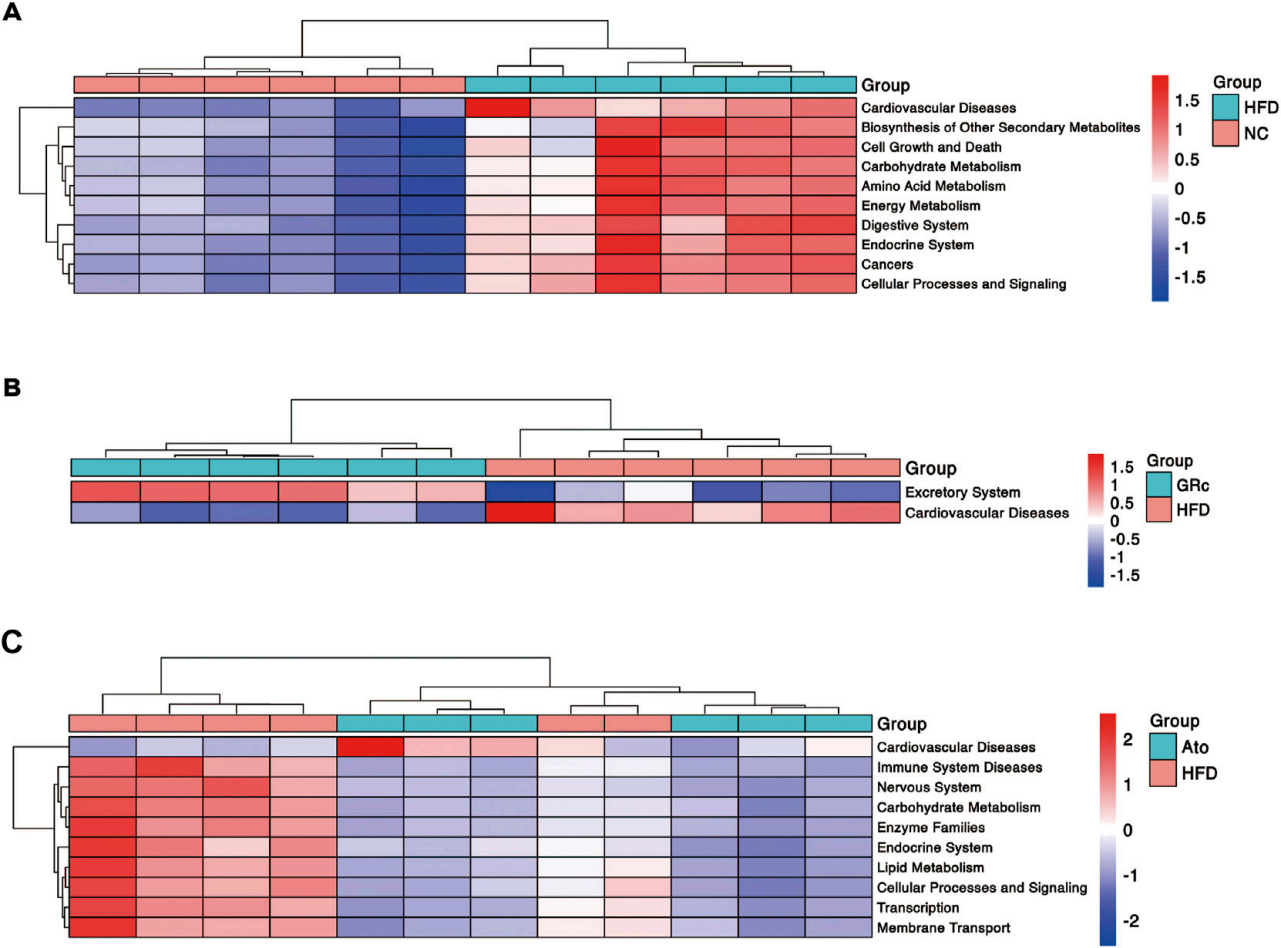
Predictive functional profiling of microbial communities by PICRUSt2 (*n* = 6). **(A)** Differences in intestinal microbiome functional profiling between HFD and NC groups. **(B)** Differences in intestinal microbiome functional profiling between GRc and HFD groups. **(C)** Differences in intestinal microbiome functional profiling between Ato and HFD groups. NC, normal control; HFD, high-fat diet; Ato, atorvastatin; GRc, ginsenoside Rc.

Functional profiling of microbial communities in level 2 of KEGG pathways was predicted by PICRUSt2. When fed with HFD, the microbiome in mice had significantly more functional genes for CVDs, biosynthesis of other secondary metabolites, cell growth and death, carbohydrate metabolism, amino acid metabolism, endocrine system, digestive system, energy metabolism, cancers, cellular processes, and signaling (Figure 6A). Compared with the HFD group, the GRc group contained significantly less functional genes for CVDs, implying that microbial communities in the GRc group might have a protective role in CVDs (Figure 6B). After Ato treatment, the microbiome in mice had significantly less functional genes for immune system diseases, nervous system, carbohydrate metabolism, enzyme families, endocrine system, lipid metabolism, cellular processes and signaling, transcription, and membrane transport, while microbiome genes for CVDs tended to increase (Figure 6C).

### Ginsenoside Rc modulated the fecal metabolites in ApoE^−/−^ mice

**FIGURE 7:**
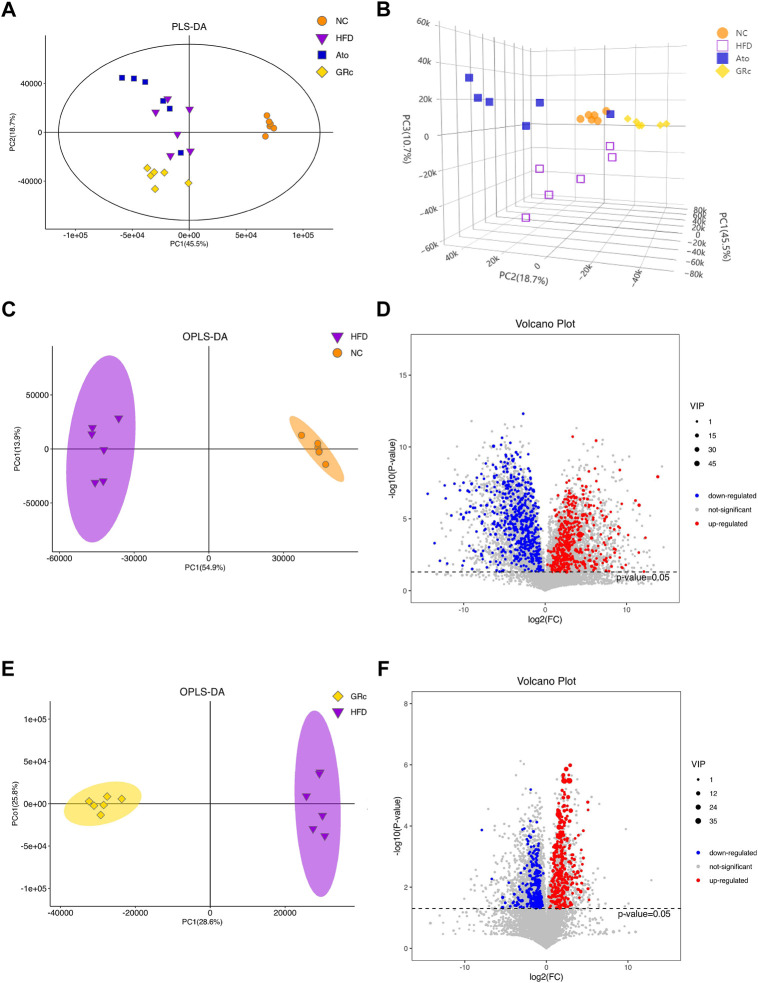
GRc modulated the fecal metabolites in ApoE^−/−^ mice (*n* = 6). **(A)** Two-dimensional PLS-DA score plots per group. **(B)** Three-dimensional PLS-DA score plots per group. **(C)** OPLS-DA score plot in NC and HFD groups. **(D)** Relative volcano plot in NC and HFD groups. **(E)** OPLS-DA score plot in GRc and HFD groups. **(F)** Relative volcano plot in GRc and HFD groups. NC, normal control; HFD, high-fat diet; Ato, atorvastatin; GRc, ginsenoside Rc.

The PLS-DA and OPLS-DA models were used to confirm the differences in fecal metabolites in groups. Seven-fold cross-validation and 200 response permutation testing were applied to prevent overfitting, and the results showed good quality of the models (Supplementary Figure S4). The PLS-DA plots showed an excellent separation among the NC, HFD, and GRc groups, while there was no obvious separation between Ato and HFD groups (Figures 7A,B). In addition, the OPLS-DA plots between HFD and NC groups also exhibited apparent differences (Figure 7C), implying that there was a significant difference in fecal metabolites between HFD and NC groups. Then, differential metabolites between HFD and NC groups were selected with the criteria of VIP > 1 and *p* < 0.05. Compared with the NC group, there were 510 significantly upregulated and 605 downregulated metabolites in the HFD group (Figure 7D). Expectedly, the OPLS-DA plots between GRc and HFD groups showed clear separation (Figure 7E). In the GRc group, 307 metabolites were significantly upregulated and 339 metabolites were downregulated in HFD-induced ApoE^−/−^ mice (Figure 7F). These results implied that GRc could modulate fecal metabolic profiles in ApoE^−/−^ mice fed with HFD.

### Ginsenoside Rc regulated the metabolic pathways related to atherosclerosis

**FIGURE 8:**
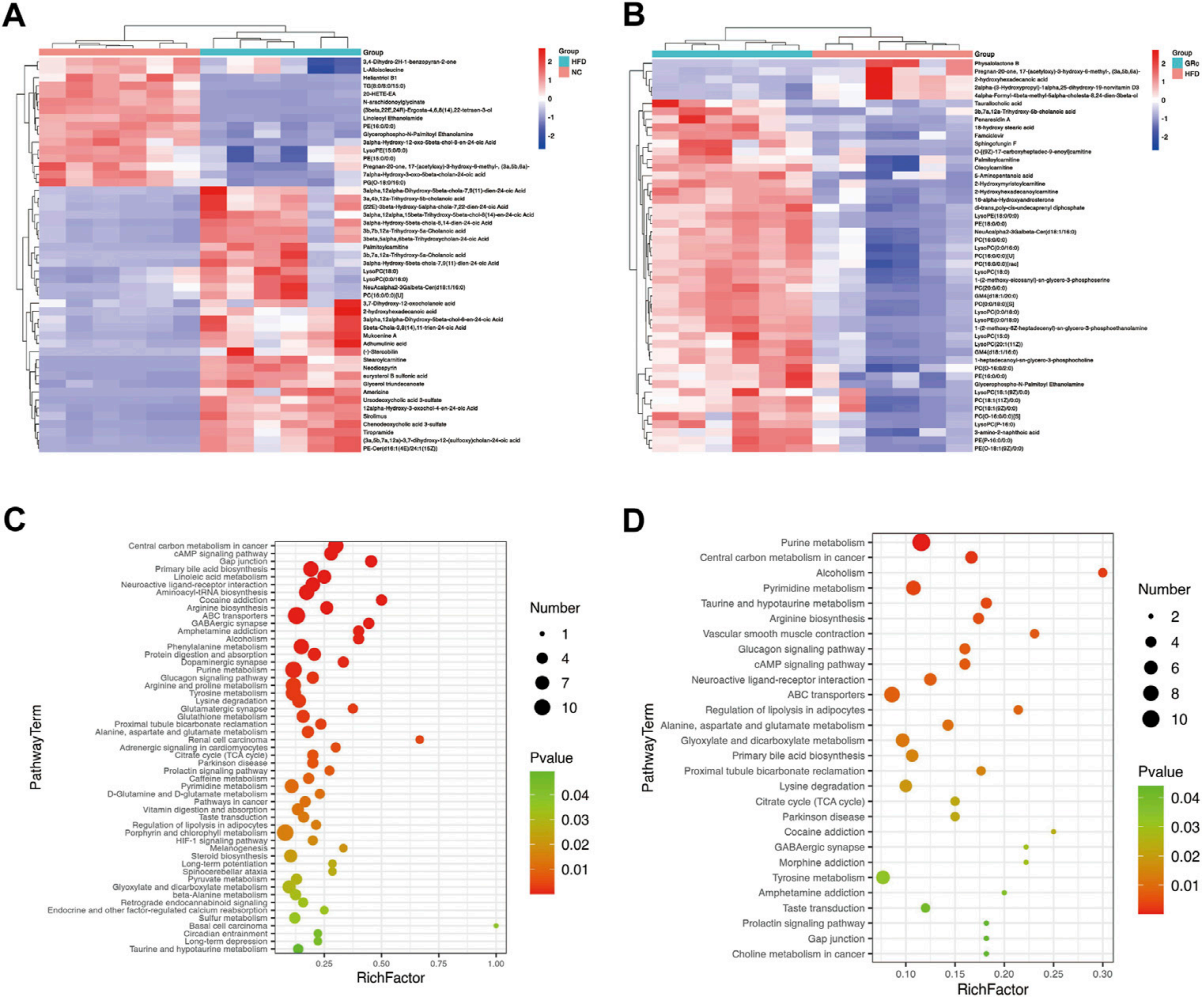
GRc regulated the metabolic pathways in ApoE^−/−^ mice (*n* = 6). **(A)** Clustering heatmap of differential metabolites in feces between HFD and NC groups. **(B)** Clustering heatmap of differential metabolites in feces between GRc and HFD groups. **(C)** KEGG pathway enrichment scatterplot between HFD and NC groups. **(D)** KEGG pathway enrichment scatterplot between GRc and HFD groups. Rich factor indicates the number of differentially expressed metabolites located in the KEGG/the total number of metabolites located in the KEGG (NC, normal control; HFD, high-fat diet; GRc, ginsenoside Rc).

To display the differences in the fecal profile clearly, top 50 differential metabolites between HFD/NC and GRc/HFD were clustered and are shown in Figures 8A,B. Then, KEGG pathway enrichment analysis was performed based on the whole differentially expressed metabolites. Notably, the significantly different metabolites between NC and HFD groups were enriched in 53 significant pathways (*p* < 0.05) (Figure 8C), and the differential metabolites between HFD and GRc groups were enriched in 28 significant pathways (*p* < 0.05) (Figure 8D). There were 23 overlapped pathways between NC/HFD and HFD/GRc, including purine metabolism, tricarboxylic acid (TCA) cycle, taurine and hypotaurine metabolism, arginine biosynthesis, glucagon signaling pathway, ATP-binding cassette (ABC) transporters, and primary bile acid biosynthesis, which were related to AS. These pathways might be potential pathogenesis mechanisms and therapeutic targets (Table 1), and the differential metabolites from the aforementioned seven pathways were investigated. In the purine metabolism pathway, adenine and uric acid were apparently increased in the HFD group and significantly decreased in the GRc group, and the same trend appeared in the levels of malic acid, fumaric acid, oxoglutaric acid, citric acid in the TCA cycle, and glucagon signaling pathways. In the taurine and hypotaurine metabolism pathway, taurine and L-glutamate in the HFD group were lower than those in the NC group, and these metabolites were apparently increased after GRc treatment; the same trend was detected in citrulline, ornithine, L-glutamate, and L-glutamine in the arginine biosynthesis pathway and sorbitol, allose, lysine, and hydroxyproline in the ABC transporter pathway. In the primary bile acid biosynthesis pathway, cholesterol and glycochenodeoxycholic acid (GCDCA) were higher, while the levels of cholic acid (CA), chenodeoxycholic acid (CDCA), glycine, isolithocholic acid (isoLCA), taurochenodeoxycholic acid (TCDCA), and taurocholic acid (TCA) were lower in the HFD group than those in the NC group. These metabolites were significantly reversed in the GRc group (Supplementary Tables S1, S2).

**TABLE 1:** Potential metabolite pathway regulated by GRc in the AS model.

No.	KEGG pathway	HFD/NC		GRc/HFD	
		Rich factor	*p*-value	Rich factor	*p*-value
**1**	Purine metabolism	0.1158	0.000829	0.1158	0.000136
**2**	TCA cycle	0.2000	0.006123	0.1500	0.022725
**3**	Taurine and hypotaurine metabolism	0.1364	0.049406	0.1818	0.004204
**4**	Arginine biosynthesis	0.2608	0.000163	0.1739	0.004969
**5**	Glucagon signaling pathway	0.2000	0.002161	0.1600	0.006768
**6**	ABC transporters	0.1290	0.000170	0.0860	0.007640
**7**	Primary bile acid biosynthesis	0.1915	0.000005	0.1064	0.014404

### Correlation between gut metabolism and atherosclerosis

**FIGURE 9:**
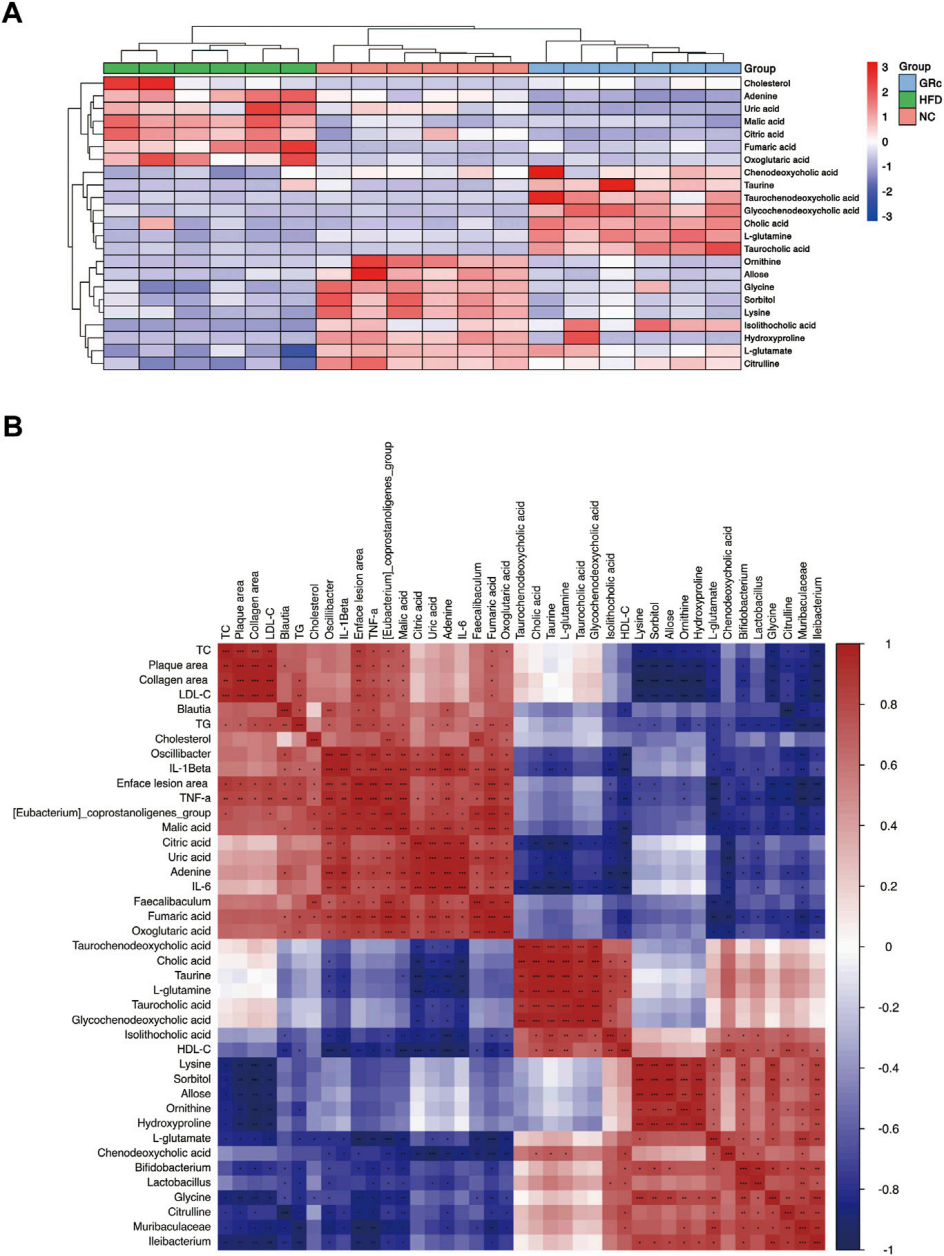
Correlation between gut metabolism and atherosclerosis. **(A)** Clustering heatmap of potential biomarkers in feces. **(B)** Spearman correlation analysis among gut microbiota, differentially expressed fecal metabolites, and atherosclerotic parameters. The correlation analysis value is represented by the colors of grinds. Red represents positive correlation, and blue represents negative correlation. *p < 0.05, **p < 0.01, and ***p < 0.001 (NC, normal control; HFD, high-fat diet; GRc, ginsenoside Rc).

To further understand the metabolic difference, we clustered the heatmap of potential biomarkers in purine metabolism, TCA cycle, taurine and hypotaurine metabolism, arginine biosynthesis, glucagon signaling pathway, ABC transporters, and primary bile acid biosynthesis pathways. As shown in Figure 9A, potential biomarkers in feces were well clustered in different groups. Compared with the HFD group, differentially expressed fecal metabolites in the GRc group were generally closer to the NC group, implying that gut metabolism was more similar to the GRc group than the NC group. Spearman correlation was examined for the possible connection among gut microbiota, potential biomarkers in feces, and atherosclerotic indexes.

We first investigated the relationship between gut microbiota and fecal potential biomarkers. *Blautia* was positively correlated with adenine and negatively correlated with citrulline. The *Eubacterium_coprostanoligenes_group* had a positive correlation with fumaric acid, malic acid, citric acid, oxoglutaric acid, adenine, uric acid, and cholesterol, with a negative correlation with L-glutamate, citrulline, glycine, and CDCA. *Faecalibaculum* was positively correlated with malic acid, citric acid, adenine, uric acid, and cholesterol while negatively correlated with L-glutamate and CDCA. *Oscillibacter* was positively correlated with fumaric acid, malic acid, citric acid, oxoglutaric acid, adenine, and uric acid, with a negative correlation with taurine, L-glutamate, citrulline, and isoLCA. Then, *Bifidobacterium* was positively correlated with L-glutamate, ornithine, isoLCA, and glycine while negatively correlated with fumaric acid, malic acid, oxoglutaric acid, adenine, uric acid, and cholesterol. *Lactobacillus* was positively correlated with L-glutamate, sorbitol, allose, lysine, hydroxyproline, and isoLCA and negatively correlated with fumaric acid, malic acid, citric acid, adenine, and uric acid. Muribaculaceae had a positive correlation with L-glutamate, citrulline, ornithine, sorbitol, lysine, hydroxyproline, and glycine, with a negative correlation with fumaric acid, malic acid, oxoglutaric acid, adenine, and uric acid. Finally, *Ileibacterium* was positively correlated with L-glutamate, citrulline, ornithine, L-glutamine, sorbitol, lysine, hydroxyproline, and glycine while negatively correlated with fumaric acid, malic acid, and oxoglutaric acid. Above all, *Blautia*, *(Eubacterium)_coprostanoligenes_group*, *Faecalibaculum*, and *Oscillibacter* seemed to have a positive correlation with metabolites in the glucagon signaling pathway, TCA cycle pathway (fumaric acid, malic acid, citric acid, and oxoglutaric acid), and purine metabolism (adenine and uric acid) and a negative correlation with metabolites in taurine and hypotaurine metabolism (taurine and L-glutamate), arginine biosynthesis (citrulline, ornithine, and L-glutamine), ABC transporter pathway (sorbitol, allose, lysine, and hydroxyproline), and primary bile acid biosynthesis (CDCA, isoLCA, and glycine), while *Bifidobacterium*, *Lactobacillus*, Muribaculaceae, and *Ileibacterium* showed opposite changes.

Regarding the relationship between fecal potential biomarkers and atherosclerotic indexes, almost metabolites in the glucagon signaling pathway and TCA cycle pathway (fumaric acid, malic acid, citric acid, and oxoglutaric acid) were positively correlated with atherosclerotic indexes except the HDL-C level, and uric acid and adenine in the purine metabolism pathway were positively correlated with enface lesion area and serum levels of TG, IL-6, IL-1β, and TNF-α. Metabolites in taurine and hypotaurine metabolism (L-glutamate) and arginine biosynthesis (citrulline and ornithine) were negatively correlated with atherosclerotic parameters except the HDL-C level, and metabolites in ABC transporters (sorbitol, allose, lysine, and hydroxyproline) negatively correlated with atherosclerotic parameters except serum levels of HDL-C, IL-6, and IL-1β. Furthermore, fecal potential biomarkers in the primary bile acid biosynthesis (CA, CDCA, TCA, TCDCA, GCDCA, isoLCA, and glycine) pathway had a negative correlation mainly with inflammatory factors (Figure 9B). These results implied that metabolites in purine metabolism, TCA cycle, and glucagon signaling pathways positively correlated with atherosclerotic injury, while those in taurine and hypotaurine metabolism, arginine biosynthesis, ABC transporters, and primary bile acid biosynthesis pathways were negatively correlated with atherosclerotic injury. In general, there was an intensive connection among gut microbiota, differentially expressed fecal metabolites, and AS**.**

## Discussion

In the present study, we investigated the impact and potential mechanism of GRc against AS in HFD-induced ApoE^−/−^ mice. The changes in serum lipid and inflammatory cytokines, compositional structure of gut microbiota, fecal metabolites, and KEGG enrichment pathways were observed after treatment with GRc. In addition, the correlation between gut metabolism and AS was determined. We found that GRc markedly alleviated atherosclerotic plaque in ApoE^−/−^ mice. Meanwhile, GRc treatment not only improved serum lipid disorder and systematic inflammation but also increased the diversity and richness of gut microbiota.In addition, GRc treatment regulated specific gut microbiota, fecal metabolites, and relative metabolic pathways related to CVDs. Moreover, differential fecal metabolites between groups also showed a strong correlation with differential gut microbiota and atherosclerotic injury.

The elevated plasma LDL-C concentration is a major risk factor for atherosclerotic CVDs. Lowering serum lipid permitted the initiation and the progression of AS (Khatana et al., 2020). In contrast, plasma HDL-C concentrations exert a protective influence on inflammation, oxidation, angiogenesis, and glucose homeostasis and have favorable effects on AS (Nicholls and Nelson, 2019). The current study demonstrated that GRc treatment significantly reduced the plasma levels of TC, TG, and LDL-C and increased HDL-C levels in HFD-induced ApoE^−/−^ mice, which has been frequently used as the predominant animal model of AS. Apart from lipid disorders, the convincing evidence indicated that AS is a chronic inflammatory disease, characterized by elevated inflammatory cytokines such as TNF-α, IL-1β, and IL-6 in serum or atherosclerotic plaque (Libby et al., 2018). Our results indicated that serum levels of TNF-α, IL-1β, and IL-6 were markedly decreased after GRc treatment. Moreover, atherosclerotic plaque in the aorta, atherosclerotic lesions, and collagen content in the aortic sinus were significantly ameliorated in the GRc group. Similar results were found in the Ato group, implying that both GRc and Ato had anti-atherosclerotic effect.

In recent years, remarkable attention has been attracted toward the gut and its accompanying microbial communities due to their important roles in physiological and pathological events in the host (Andoh, 2016). Increasing studies indicated that gut microbiota changes are associated with numerous disease states including CVDs (Witkowski et al., 2020). It has been reported that reduced diversity and richness of gut microbial species increased the risk of developing AS (Menni et al., 2018). Our study was consistent with previous results that the HFD decreased the diversity of gut microbiota (Han et al., 2020), and GRc treatment significantly increased the diversity and richness of microbial species in ApoE^−/−^ mice. Consistent with a previous study (Kim et al., 2019; SudunLiu et al., 2019), Ato treatment had no obvious effects on diversity and richness of microbial species. In addition, the altered composition of the bacterial communities in different specific microbial species at different taxonomic levels also had an intensive connection with CVDs. Firmicutes and Bacteroidetes are two dominant phyla in the composition of human gut microbiota. An increased Firmicutes to Bacteroidetes ratio was reported to be positively associated with CVDs (De Filippis et al., 2016; Emoto et al., 2016). Firmicutes deteriorated metabolic endotoxins and inflammation and increased the risk of obesity and AS, while Bacteroidetes had an opposite impact (Abdallah Ismail et al., 2011; Dong et al., 2019). PCoA analysis in our results showed that there was much more significance in the composition of gut microbiota in NC, HFD, and GRc groups, in which relative abundances of gut microbiota at phylum and genus levels also showed a marked difference. GRc treatment markedly increased the abundance of Bacteroidetes and decreased that of Firmicutes and then contributed to a decrease in the Firmicutes to Bacteroidetes ratio which was higher in the HFD group. In accordance with previous studies (Kim et al., 2019; Zimmermann et al., 2020), Ato treatment significantly increased the abundance of Bacteroidetes and decreased Firmicutes in HFD-induced mice. Emerging evidence suggested that bacteria of Deferribacteraceae and *Eubacterium_coprostanoligenes_group* are correlated with trimethylamine/trimethylamine N-oxide production, which was considered a risk marker for the development of AS into CVDs (Koeth et al., 2013; Rath et al., 2017), while Lachnospiraceae was associated with the lower trimethylamine N-oxide level and anti-thrombotic phenotype (Zhu et al., 2016). On the other hand, a high level of *Eubacterium_coprostanoligenes_group* was linked with obesity, which is a risk factor for AS (Gomes et al., 2018). Furthermore, both *Lactobacillus* and *Bifidobacterium* are widely reported beneficial to human health. *Lactobacillus acidophilus* modulated oxidative stress, inflammatory process (Chen et al., 2013), and lipid metabolism via regulating reverse cholesterol transport in HFD-induced ApoE^−/−^ mice (Huang et al., 2014; Zhao et al., 2019), while *Bifidobacterium* displayed an effect of decreasing visceral fat mass in people (Rath et al., 2017), subsequently ameliorated the progression of AS, and mitigating effects on HFD-induced obesity was accompanied with an increased Muribaculaceae level (Ohue-Kitano et al., 2019). The 16S rRNA analysis in our experiment showed that the family of Deferribacteraceae and *Eubacterium*_*coprostanoligenes*_group were significantly increased and Muribaculaceae, Lachnospiraceae, Bifidobacteriaceae, and Lactobacillaceae were markedly decreased in the HFD group. GRc treatment reversed the effect on gut microbiota at the family level induced by HFD, while Ato treatment only reversed the levels of Muribaculaceae and *Eubacterium*_*coprostanoligenes*_group induced by HFD. At the genus level, the studies showed that elevated abundance of *Oscillibacter* was closely related to gut permeability and inflammation in obese mice (Lam et al., 2012), while *Ileibacterium* protected mice from adiposity (Den Hartigh et al., 2018). Our investigation showed that GRc significantly increased the relative abundance of *Lactobacillus*, *Bifidobacterium*, *Ileibacterium*, and Muribaculaceae, with a decreased relative abundance of *Oscillibacter*, *Eubacterium_coprostanoligenes_group*, *Blautia*, and *Faecalibacterium*, while Ato treatment had a similar effect to GRc without significantly reversing levels of *Lactobacillus*, *Bifidobacterium*, and *Ileibacterium* induced by HFD. The results of *Blautia* and *Faecalibacterium* in our study were inconsistent with previous studies that showed *Blautia* was reduced in HFD-induced mice (Wu et al., 2020) and *Faecalibacterium* exhibited cardio-protective effects (Gerdes et al., 2020), while some other intestinal floras including *Bacteriodes* and *Parabacteriodes* were reduced by GRc treatment in our study, which might potentially influence the AS progression and would worth further study. In addition to the composition of the intestinal flora, the whole function of microbial communities receives increasing attention (Whidbey and Wright, 2019). Predictive functional profiling of microbial communities by PICRUSt2 showed that GRc significantly reduced functional genes for CVDs, while Ato tended to enhance functional genes for CVDs. Above all, GRc might exhibit anti-atherosclerotic effects by regulating the gut microbiota related to AS.

Metabolomics has been routinely applied as a tool for the discovery of biomarkers, system-level effects of metabolites, and subtle alterations in biological pathways, which might provide insights into the mechanisms underlying various diseases (Johnson et al., 2016). PLS-DA and OPLS-DA analyses in our study showed significantly different fecal metabolic phenotypes between NC/HFD and GRc/HFD. Then, fecal differential metabolites between groups were clustered and annotated in KEGG pathways. Accumulating evidence showed an intensive connection between metabolites of gut microbiota and CVDs. Uric acid and adenine are end products of purine metabolism in humans. An intensive connection has been shown between elevated uric acid and CVDs, and the mechanisms underlying deleterious effects of elevated uric acid on cardiovascular health included increased oxidative stress, reduced availability of nitric oxide and endothelial dysfunction, promotion of local and systemic inflammation, vasoconstriction and proliferation of vascular smooth muscle cells, insulin resistance, and metabolic dysregulation (Ndrepepa, 2018). Our results showed that high levels of uric acid and adenine were induced by HFD, while GRc markedly reduced uric acid and adenine in the purine metabolism pathway. Recent studies have shown that mitochondrial metabolites in the TCA cycle were not only considered mere intermediate substrates for energy generation but also acted as key signaling molecules regulating gene transcription and translation. Succinate has emerged as a circulating biomarker for several metabolic and CVDs (Pell et al., 2016; Serena et al., 2018; Ceperuelo-Mallafre et al., 2019). On the other hand, elevated malic acid and citric acid were associated with a higher risk of CVDs (Bullo et al., 2021), and fumaric acid accumulation activated gene transcription for TNF-α and IL6 cytokines and increased ROS signaling via binding to glutathione (Blatnik et al., 2008; Zheng et al., 2015). In our study, we found that HFD significantly increased the metabolites in the TCA cycle or glucagon signaling pathways, such as malic acid, fumaric acid, oxoglutaric acid, and citric acid. GRc markedly decreased aforementioned metabolites. Recently, a strong relationship between alterations in amino acid metabolism and the development of AS had been observed, in which L-arginine and its metabolism improved NO-dependent vasodilator function and reduced atherosclerotic plaques with reversed endothelial dysfunction (Zaric et al., 2020). In our study, GRc did not significantly influence the L-arginine level but increased the intermediate substrates for arginine biosynthesis, including citrulline, ornithine, L-glutamate, and L-glutamine, which might accelerate the biosynthesis and utilization of L-arginine. In addition, GRc increased sorbitol, allose, lysine, and hydroxyproline in the composition of ABC transporters which were widely considered receptors of reverse cholesterol transport with an important role in the prevention and treatment of AS (Kotlyarov and Kotlyarova, 2021). Taurine was considered to improve antioxidant effects and lipid profile and reduce atherosclerotic lesion formation. The hypocholesterolemic effects of taurine are mediated by enhanced cholesterol degradation and the excretion of bile acid (Zaric et al., 2020). Bile acid is an important signaling molecule and metabolic regulator of lipid, glucose, and energy metabolism in AS (Chiang et al., 2020). Fecal excretion of bile acid is a major rink for cholesterol, and bile acids lost in the process need to be replaced by *de novo* synthesis from cholesterol. Furthermore, bile acids have also been shown to influence host lipid metabolism through the farnesoid X receptor (FXR) and the Takeda G-protein-coupled bile acid receptor (TGR) in the intestine and liver. Primary bile acids such as CA and CDCA and the secondary bile acids such as lithocholic acid (LCA) and deoxycholic acid (DCA) are FXR agonists, while LCA and DCA act as agonists to TGR5 (Schoeler and Caesar, 2019). Activation of FXR and TGR5 promoted energy metabolism and significantly reduced atherosclerotic formation (Miyazaki-Anzai et al., 2018). Our results showed that cholesterol was reduced, and the levels of taurine, CA, CDCA, glycine, isoLCA, TCDCA, and TCA were increased upon GRc treatment. An increase in primary bile acids and secondary bile acids might accelerate the enterohepatic circulation and fecal excretion of bile acids and activation of FXR and/or TGR5 and then hasten cholesterol metabolism, given that most isoLCA is excreted into feces and small amounts reabsorbed to the liver. Consequently, GRc treatment markedly regulated fecal metabolites in HFD-induced ApoE^−/−^ mice. The underlying mechanism of GRc relieving AS might result from improving CVD-related metabolic pathways, in which differential metabolites were annotated. These results to some extent proved the predictive functional profiling of microbial communities by PICRUSt2. Taken together, fecal differential metabolites mentioned previously might be potential biomarkers of GRc treatment against AS in ApoE^−/−^ mice.

Previous studies reported that *Eubacterium_coprostanoligenes_group* (Gomes et al., 2018) and *Oscillibacter* (Lam et al., 2012) could aggravate CVDs, while *Lactobacillus*, *Bifidobacterium*, Muribaculaceae, and *Ileibacterium* exerted protective effects (Huang et al., 2014; Rath et al., 2017; Den Hartigh et al., 2018; Ohue-Kitano et al., 2019; Zhao et al., 2019), implying that *Lactobacillus*, *Bifidobacterium*, Muribaculaceae, and *Ileibacterium* were beneficial intestinal floras to cardiovascular health, while *Eubacterium_coprostanoligenes_group* and *Oscillibacter* played the opposite roles. After GRc treatment, atherosclerotic injury in HFD-induce mice was alleviated.In addition, beneficial intestinal floras to cardiovascular health were increased and harmful ones were decreased, accompanied by increased fecal differential metabolites in taurine and hypotaurine metabolism (taurine and L-glutamate), arginine biosynthesis (citrulline, ornithine, and L-glutamine), ABC transporters (sorbitol, allose, lysine, and hydroxyproline), primary bile acid biosynthesis (CA, CDCA, TCA, TCDCA, GCDCA, isoLCA, and glycine), and decreased metabolites in purine metabolism (adenine and uric acid), TCA cycle, and glucagon signaling pathway (fumaric acid, malic acid, citric acid, and oxoglutaric acid). A correlation between gut metabolism and AS was investigated. Adenine and uric acid in the purine metabolism pathway had a positive correlation with intestinal floras, namely, *Eubacterium_coprostanoligenes_group*, *Faecalibaculum*, and *Oscillibacter*, and AS indexes (enface lesion area, TG, and inflammatory factors) while negatively correlated with intestinal floras, namely, *Lactobacillus*, *Bifidobacterium*, and Muribaculaceae, which were consistent with previous studies that uric acid and adenine aggravate AS (Ndrepepa, 2018).

Of note, previous reports indicated that fumaric acid accumulation could accelerate inflammation (Zheng et al., 2015). Malic acid and citric acid might be risk factors for AS (Bullo et al., 2021). Our study showed that fumaric acid, citric acid, and malic acid in the TCA cycle were positively correlated with gut microbiota (*Eubacterium_coprostanoligenes_group*, *Faecalibacterium*, and *Oscillibacter*) and AS indexes and negatively correlated with intestinal floras (*Lactobacillus*, *Bifidobacterium*, Muribaculaceae, and *Ileibacterium*). Metabolites in the ABC transporter pathway were positively correlated with intestinal floras (*Lactobacillus*, Muribaculaceae, and *Ileibacterium*) and negatively connected with AS indexes (enface lesion area, plaque area, collagen area, TC, TG, LDL-C, and TNF-α). Moreover, citrulline in arginine biosynthesis was negatively correlated with gut microbiota (*Eubacterium_coprostanoligenes_group* and *Oscillibacter*) and AS indexes, while it was positively connected with Muribaculaceae and *Ileibacterium*. Ornithine was negatively correlated with AS indexes (enface lesion area, plaque area, collagen area, TC, TG, LDL-C, and TNF-α) while positively correlated with intestinal floras (*Bifidobacterium*, Muribaculaceae, and *Ileibacterium*) in our study, supporting that L-arginine and its metabolism could improve AS (Zaric et al., 2020). Furthermore, increased bile acids might accelerate the enterohepatic circulation and fecal excretion of bile acids and activation of FXR and/or TGR5 and then hasten cholesterol metabolism and improve AS (Miyazaki-Anzai et al., 2018; Schoeler and Caesar, 2019; Chiang et al., 2020). A correlation among gut microbiota, bile acids, and AS indexes was in good agreement with the studies mentioned previously, and CDCA was negatively correlated with gut microbiota (*Eubacterium_coprostanoligenes_group* and *Faecalibacterium*) and AS indexes (enface lesion area, TG, and inflammatory factors), while it was positively connected with HDL-C. Glycine showed a negative correlation with *Eubacterium_coprostanoligenes_group* and AS indexes, with a positive correlation with intestinal floras (*Bifidobacterium*, Muribaculaceae, and *Ileibacterium*). Notably, isoLCA was negatively correlated with *Oscillibacter* and AS indexes (enface lesion area and inflammatory factors) while positively correlated with *Lactobacillus*, *Bifidobacterium*, and HDL-C levels. The altered intestinal floras, fecal differential metabolites mentioned previously, and atherosclerotic injury showed an intensive correlation. *Eubacterium_coprostanoligenes_group*, *Faecalibaculum*, *Oscillibacter*, and *Blautia* enriched in the HFD group, indicating a positive correlation with the differential metabolites that were positively correlated with atherosclerotic injury and negatively correlated with a negative regulator of atherosclerotic injury. *Ileibacterium*, *Lactobacillus*, *Bifidobacterium*, and Muribaculaceae enriched in NC, GRc, and Ato groups with a reverse correlation. We hypothesized that GRc treatment regulated the composition of gut microbiota related to CVDs, followed by a change in fecal metabolic profiles from harmful to beneficial to cardiovascular health and finally alleviated AS. In the study, we reported the anti-atherosclerotic role of GRc in HFD-induced ApoE^−/−^ mice for the first time and applied the method of integrated analysis of 16S rRNA sequence and untargeted fecal metabolomics, which might provide a strategy for investigating other medicines. We also explored the mechanisms of GRc against AS from a novel perspective of gut metabolism. The validation of the results was absent in the study, and we will continue further experiments such as fecal transplantation in germ-free mice.

## Conclusion

In this study, GRc reduced atherosclerotic lesions, improved lipid levels, and systemic inflammation in HFD-induced ApoE^−/−^ mice without significant change in body weight. Considering the poor bioavailability of GRc in oral administration, GRc exerted an anti-atherosclerotic role probably through comprehensive effects of regulating gut microbiota and differentially expressed fecal metabolites closely related to CVDs. Our study for the first time reported the anti-atherosclerotic effect of GRc in ApoE^−/−^ mice induced with HFD. However, metabolites in serum and the role of some other intestinal floras regulated by GRc are still to be clarified.

## Data Availability

The datasets presented in this study can be found in online repositories. The names of the repository/repositories and accession number(s) can be found below: National Center for Biotechnology Information (NCBI) BioProject, http://www.ncbi.nlm.nih.gov/bioproject/874848, PRJNA874848.

## Glossary

AS: atherosclerosis
CVDs: cardiovascular diseases
GRc: ginsenoside Rc
ApoE: apolipoprotein E-deficient
NC: normal control
HFD: high-fat diet
Ato: atorvastatin
TC: total cholesterol
TG: triglyceride
LDL-C: low-density lipoprotein cholesterol
HDL-C: high-density lipoprotein cholesterol
TNF-α: tumor necrosis factor-α
IL-6: interleukin-6
IL-1β: interleukin-1β
ABC: ATP-binding cassette
TCA (cycle): tricarboxylic acid (cycle)
ORO: Oil Red O
H&E: hematoxylin and eosin
OTU: operational taxonomic unit
PCoA: principal coordinate analysis
LDA: linear discriminant analysis
LEfSe: linear discriminant analysis coupled with effect size
PICRUSt: phylogenetic investigation of communities by reconstruction of unobserved states
PCA: principal component analysis
PLS-DA: partial least-squares-discriminant analysis
VIP: variable importance in projection
KEGG: Kyoto Encyclopedia of Genes and Genomes
CA: cholic acid
CDCA: chenodeoxycholic acid
isoLCA: isolithocholic acid
TCDCA: taurochenodeoxycholic acid
TCA: tricarboxylic acid
GCDCA: glycochenodeoxycholic acid
FXR: farnesoid X receptor
TGR: Takeda G-protein-coupled bile acid receptor
LCA: lithocholic acid
DCA: deoxycholic acid
