# Supplementary material for: Ginsenoside Rc ameliorated atherosclerosis via regulating gut microbiota and fecal metabolites
Source: Front Pharmacol. 2022 Sep 15;13:990476. doi: 10.3389/fphar.2022.990476 (PMC9520581; doi:10.3389/fphar.2022.990476)
Supplement: Supplementary file 1 [file Table1.DOCX]

Supplementary Material

# Supplementary Figures


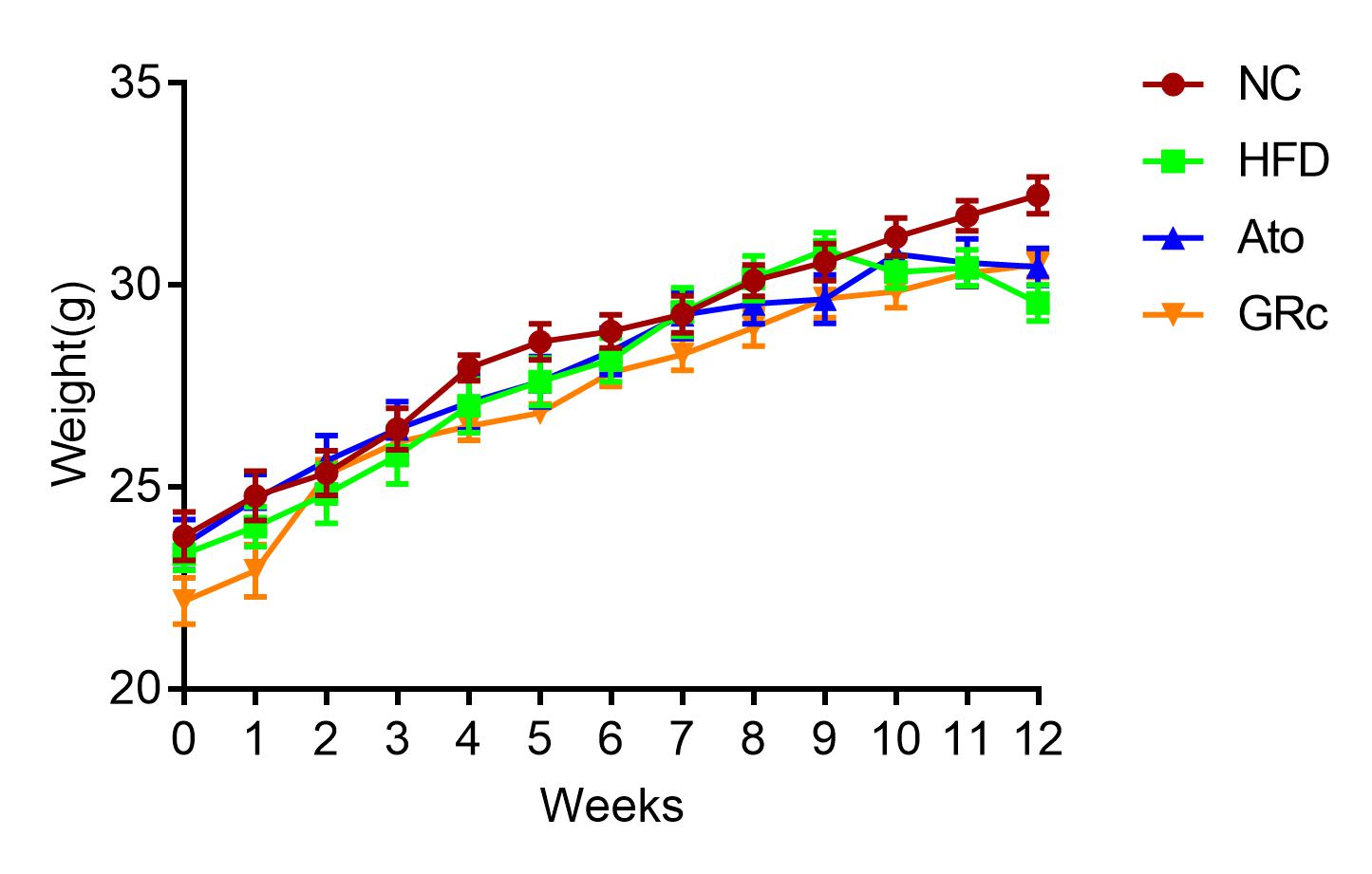


**Supplementary Figure S1.** The change of body weight during the experiment.


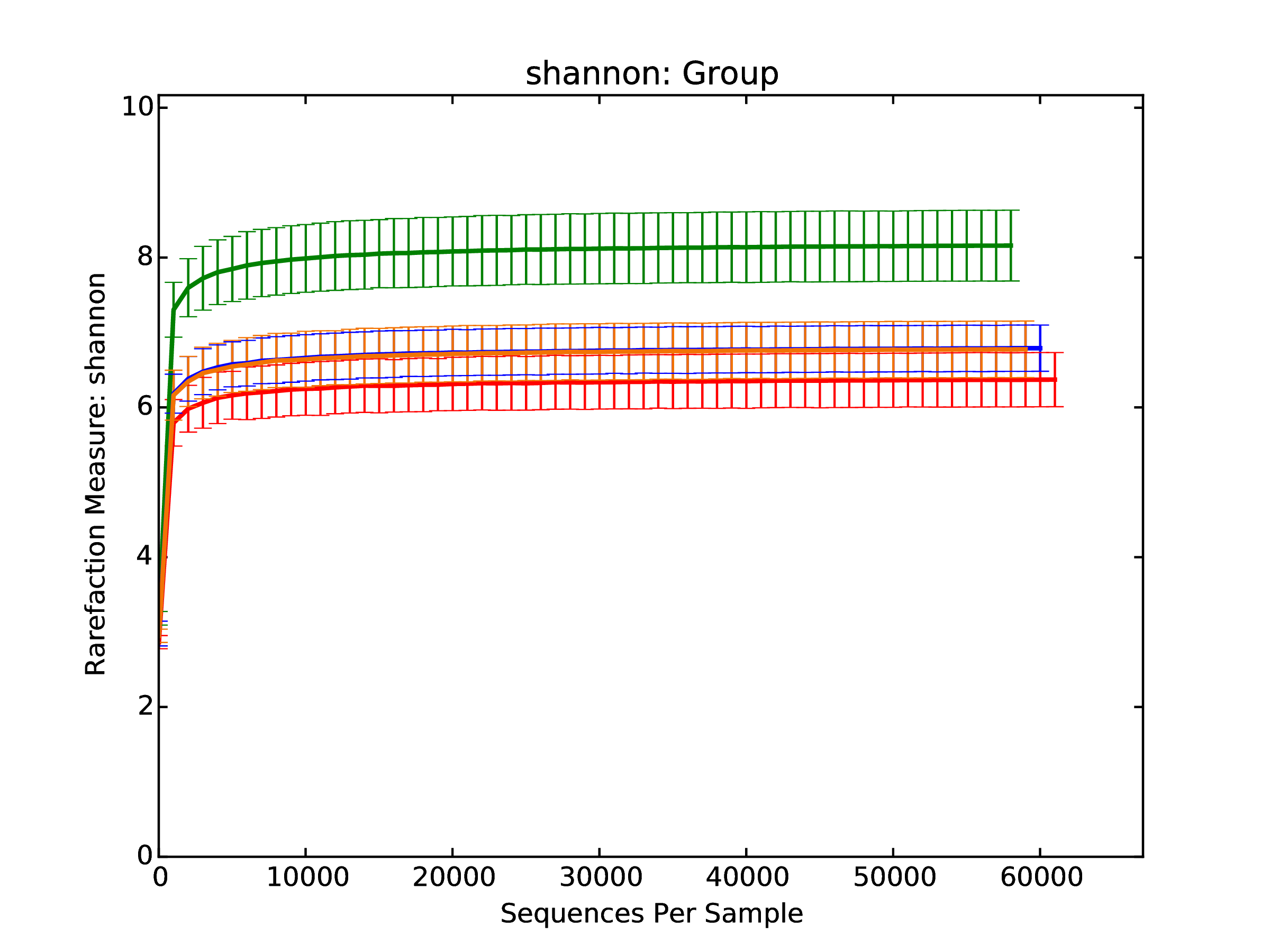


**Supplementary Figure S2.** Shannon rarefaction curve in individual groups. The abscissa indicates the number of sequencing tags to be retrieved randomly, and ordinates reflect the estimated value of the shannon index determined when a specific number of tags are retrieved.

**
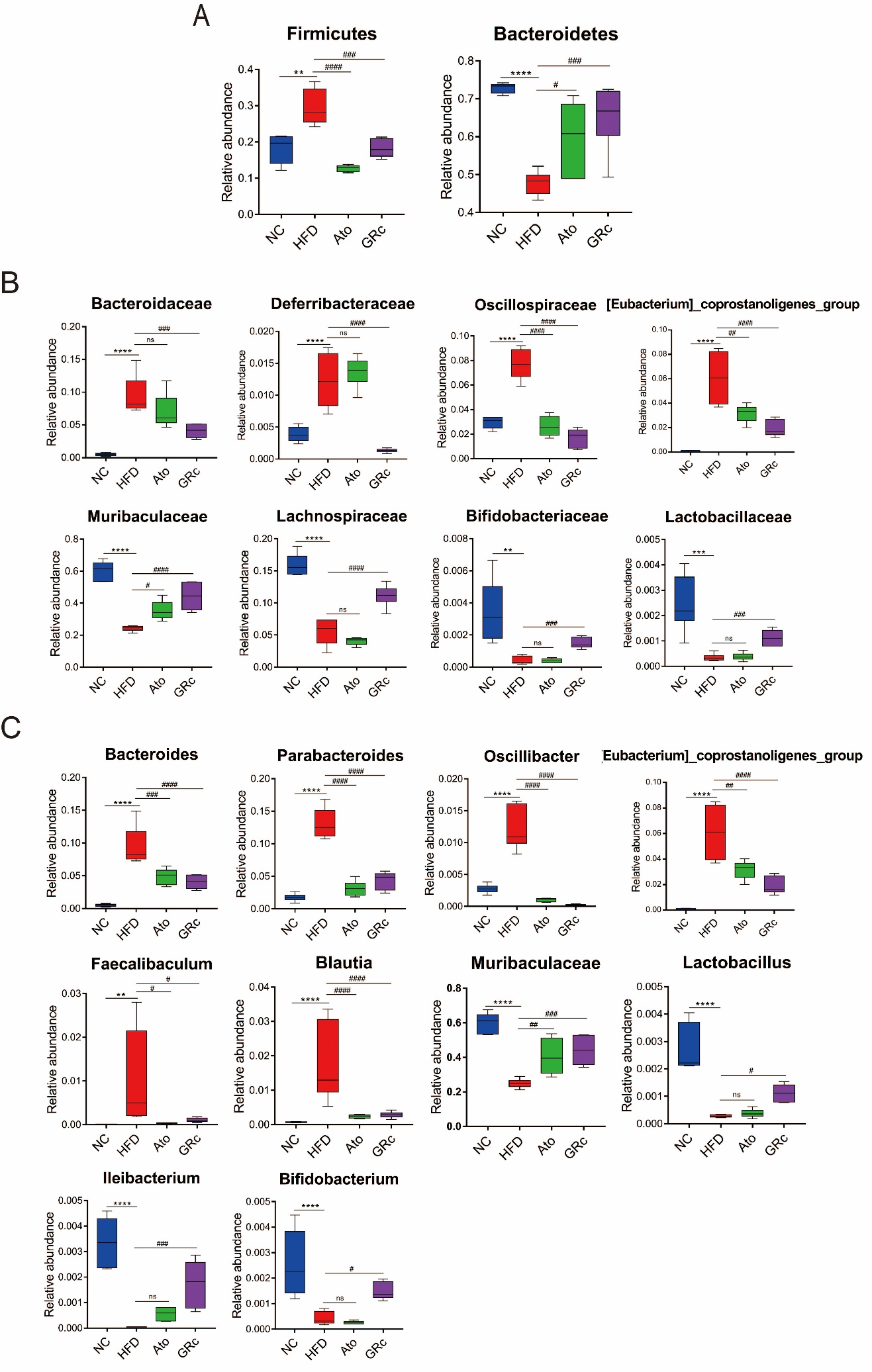
**

**Supplementary Figure S3.** GRc regulated the composition of gut microbiota at (**A**) phylum level, (**B**) family level and (**C**) genus level in HFD-induced ApoE^-/-^ mice (n=6) The data are expressed as means ± SEM. **p* < 0.05, ***p* < 0.01 and ****p* < 0.001 vs NC; #*p* < 0.05, ##*p* < 0.01 and ###*p* < 0.001 vs HFD; ns = not significant (NC = normal control; HFD = high fat diet; Ato = atorvastatin; GRc = ginsenoside Rc).


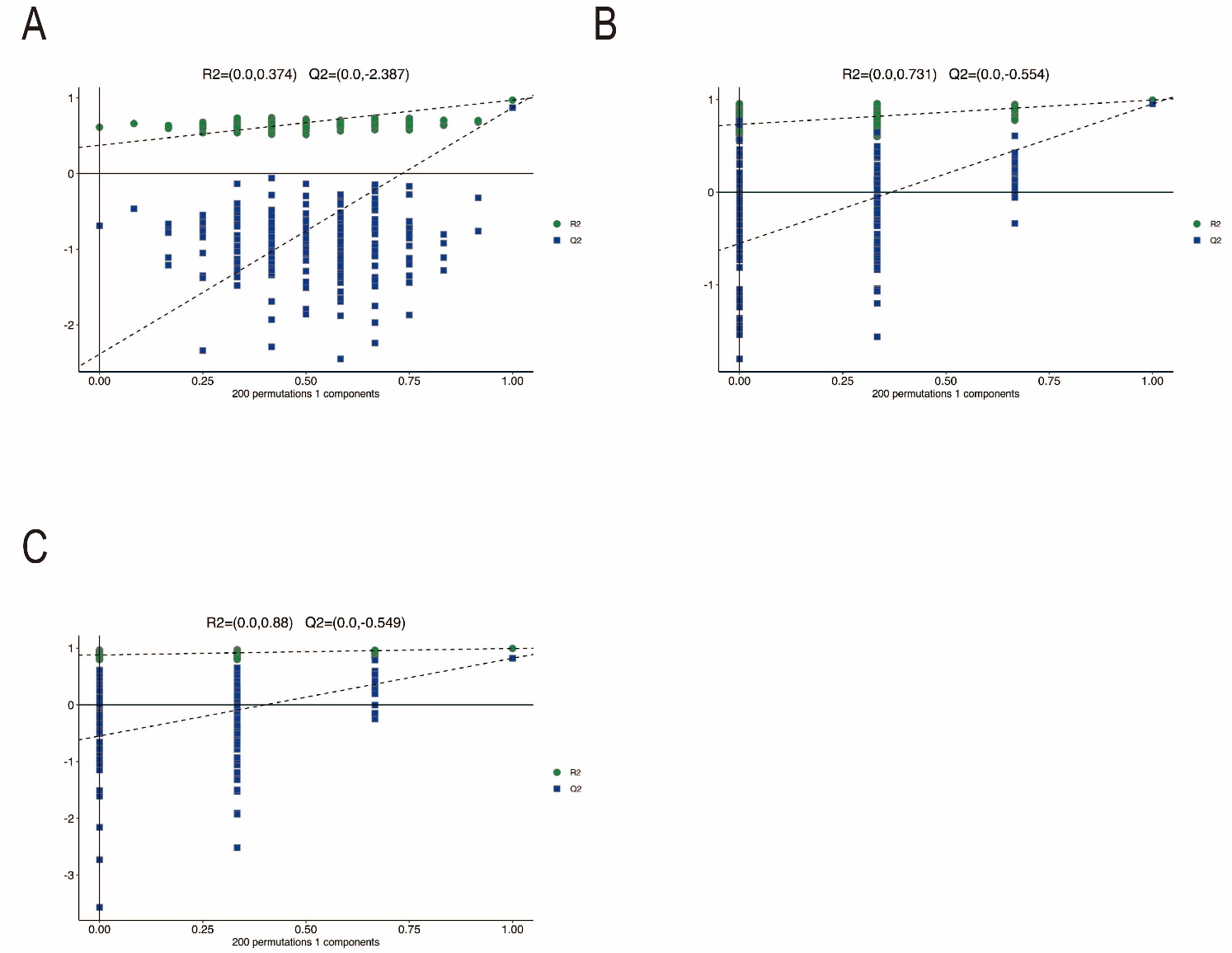


**Supplementary Figure S4.** Permutation test derived from the feces of mice in different group. (**A**) Permutation test from PLS-DA model among NC, HFD, Ato and GRc groups. (**B**) Permutation test from OPLS-DA model between NC and HFD group. (**C**) Permutation test from OPLS-DA model between GRc and HFD group. (NC = normal control; HFD = high fat diet; Ato = atorvastatin; GRc = ginsenoside Rc).

# Supplementary Tables

**Table S1** Potential biomarkers in feces associated with GRc treatment based on LC/MS analysis in HFD-induced ApoE^-/-^ mice

| NO. | RT | m/z | Formula | Metabolite | Trend | | Related pathway |
| --- | --- | --- | --- | --- | --- | --- | --- |
|  |  |  |  |  | HFD/NC | GRc/HFD |  |
| 1 | 0.7409 | 136.0620 | C_5_H_5_N_5_ | Adenine | ↑^**^ | ↓^####^ | Purine metabolism |
|  |  |  |  |  |  |  |  |
| 2 | 0.7132 | 126.0223 | C_2_H_7_NO_3_S | Taurine | ↓ | ↑^####^ | Taurine and hypotaurine metabolism; ABC transporters; Primary bile acid biosynthesis |
|  |  |  |  |  |  |  |  |
| 3 | 0.7132 | 148.0607 | C_5_H_9_NO_4_ | L-glutamate | ↓^****^ | ↑^##^ | Taurine and hypotaurine metabolism; Arginine biosynthesis |
| 4 | 0.7132 | 176.1031 | C_6_H_13_N_3_O_3_ | Citrulline | ↓^****^ | ↑^####^ | Arginine biosynthesis |
|  |  |  |  |  |  |  |  |
| 5 | 0.7132 | 147.0767 | C_5_H_10_N_2_O_3_ | L-glutamine | ↓^****^ | ↑^####^ | Arginine biosynthesis; ABC transporters |
|  |  |  |  |  |  |  |  |
| 6 | 9.6093 | 426.3215 | C_24_H_40_O_5_ | Cholic acid | ↓ | ↑^###^ | Primary bile acid biosynthesis |
| 7 | 9.809 | 375.2896 | C_24_H_40_O_4_ | Chenodeoxycholic acid | ↓^**^ | ↑^#^ | Primary bile acid biosynthesis |
| 8 | 7.8444 | 533.3260 | C_26_H_45_NO_7_S | Taurocholic acid | ↓ | ↑^##^ | Primary bile acid biosynthesis |
| 9 | 7.2267 | 498.2903 | C_26_H_45_NO_6_S | Taurochenodeoxycholic acid | ↓ | ↑^##^ | Primary bile acid biosynthesis |
| 10 | 6.9821 | 494.3120 | C_26_H_43_NO_5_ | Glycochenodeoxycholic acid | ↑ | ↑^####^ | Primary bile acid biosynthesis |
| 11 | 13.3998 | 394.3331 | C_24_H_40_O_3_ | Isolithocholic acid | ↓^****^ | ↑^###^ | Primary bile acid biosynthesis |

HFD/NC: HFD versus NC; GRc/HFD: GRc versus HFD; **p* < 0.05, ***p* < 0.01 and ****p* < 0.001 vs NC; #*p* < 0.05, ##*p* < 0.01 and ###*p* < 0.001 vs HFD (NC = normal control; HFD = high fat diet; Ato = atorvastatin; GRc = ginsenoside Rc).

**Table S2** Potential biomarkers in feces associated with GRc treatment based on GC /MS analysis in HFD-induced ApoE^-/-^ mice

| NO. | RT | Quant mass | Metabolite | Trend | | Related pathway |
| --- | --- | --- | --- | --- | --- | --- |
|  |  |  |  | HFD/NC | GRc/HFD |  |
| 1 | 21.652 | 441.2 | Uric acid | ↑^*^ | ↓^###^ | Purine metabolism |
|  |  |  |  |  |  |  |
| 2 | 11.749 | 245.1 | Fumaric acid | ↑ | ↓^##^ | Central carbon metabolism in cancer; Glucagon signaling pathway; TCA cycle |
|  |  |  |  |  |  |  |
| 3 | 13.926 | 147.1 | Malic acid | ↑^****^ | ↓^####^ | Central carbon metabolism in cancer; Glucagon signaling pathway; TCA cycle |
|  |  |  |  |  |  |  |
| 4 | 18.628 | 273.1 | Citric acid | ↑^**^ | ↓^####^ | Central carbon metabolism in cancer; Glucagon signaling pathway; TCA cycle |
|  |  |  |  |  |  |  |
| 5 | 15.274 | 198.0885 | Oxoglutaric acid | ↑^**^ | ↓^##^ | Central carbon metabolism in cancer; Glucagon signaling pathway; TCA cycle |
|  |  |  |  |  |  |  |
| 6 | 17.769 | 174.1212 | Ornithine | ↓^***^ | ↑ | Arginine biosynthesis |
| 7 | 19.952 | 319.2 | Sorbitol | ↓^****^ | ↑ | ABC transporters |
| 8 | 21.884 | 319.2 | Allose | ↓^***^ | ↑ | ABC transporters |
| 9 | 19.945 | 174.1279 | Lysine | ↓^***^ | ↑ | ABC transporters; Lysine degradation |
| 10 | 14.542 | 230.188 | Hydroxyproline | ↓^***^ | ↑ | ABC transporters |
| 11 | 28.366 | 329.3111 | Cholesterol | ↑^*^ | ↓ | Primary bile acid biosynthesis |
|  |  |  |  |  |  |  |
| 12 | 10.977 | 174.133 | Glycine | ↓^****^ | ↑ | Primary bile acid biosynthesis; Lysine degradation |

HFD/NC: HFD versus NC; GRc/HFD: GRc versus HFD; **p* < 0.05, ***p* < 0.01 and ****p* < 0.001 vs NC; #*p* < 0.05, ##*p* < 0.01 and ###*p* < 0.001 vs HFD

(NC = normal control; HFD=high fat diet; Ato = atorvastatin; GRc = ginsenoside Rc).
